# Transcriptome profiles of organ tissues from pigs experimentally infected with African swine fever virus in early phase of infection

**DOI:** 10.1080/22221751.2024.2366406

**Published:** 2024-06-07

**Authors:** Sang-Ik Oh, Sunirmal Sheet, Vuong Nghia Bui, Duy Tung Dao, Ngoc Anh Bui, Tae-Hun Kim, Jihye Cha, Mi-Rim Park, Tai-Young Hur, Young-Hun Jung, Bumseok Kim, Hu Suk Lee, Ara Cho, Dajeong Lim

**Affiliations:** a National Institute of Animal Science, Rural Development Administration, Wanju, Republic of Korea; b Laboratory of Veterinary Pathology and Biosafety Research Institute, College of Veterinary Medicine, Jeonbuk National University, Iksan, Republic of Korea; c Virology Department, National Institute of Veterinary Research, Hanoi, Vietnam; d TNT Research. Co., Ltd., R&D center, Sejong-si, Republic of Korea; e International Livestock Research Institute, Hanoi, Vietnam; f College of Veterinary Medicine, Chungnam National University, Daejoen, Republic of Korea; g Department of Animal Resources Science, College of Agriculture and Life Sciences, Chungnam National University, Daejoen, Republic of Korea

**Keywords:** African swine fever, differentially expressed gene, immune response, organ tissue tropism, transcriptome

## Abstract

African swine fever, caused by African swine fever virus (ASFV), is a highly contagious and fatal disease that poses a significant threat to the global pig industry. The limited information on ASFV pathogenesis and ASFV–host interactions has recently prompted numerous transcriptomic studies. However, most of these studies have focused on elucidating the transcriptome profiles of ASFV-infected porcine alveolar macrophages *in vitro*. Here, we analyzed dynamic transcriptional patterns *in vivo* in nine organ tissues (spleen, submandibular lymph node, mesenteric lymph node, inguinal lymph node, tonsils, lungs, liver, kidneys, and heart) obtained from pigs in the early stages of ASFV infection (1 and 3 d after viremia). We observed rapid spread of ASFV to the spleen after viremia, followed by broad transmission to the liver and lungs and subsequently, the submandibular and inguinal lymph nodes. Profound variations in gene expression patterns were observed across all organs and at all time-points, providing an understanding of the distinct defence strategies employed by each organ against ASFV infection. All ASFV-infected organs exhibited a collaborative response, activating immune-associated genes such as *S100A8*, thereby triggering a pro-inflammatory cytokine storm and interferon activation. Functional analysis suggested that ASFV exploits the PI3K-Akt signalling pathway to evade the host immune system. Overall, our findings provide leads into the mechanisms underlying pathogenesis and host immune responses in different organs during the early stages of infection, which can guide further explorations, aid the development of efficacious antiviral strategies against ASFV, and identify valuable candidate gene targets for vaccine development.

## Introduction

African swine fever, caused by the ASF virus (ASFV), is a highly contagious haemorrhagic viral disease that affects domestic pigs and wild boars of all ages, causing serious economic and production losses [1]. ASFV is a large double-stranded DNA virus that is 260–300 nm in diameter and belongs to the *Asfivirus* genus of the family *Asfarviridae* [2]. Genotype II ASFV (Georgia07) that emerged in Georgia is highly lethal [3], and a Georgia07-like virus that has an additional sequence (5′-GGAATATATA-3′) between the 173R and 1329L genes has spread to Asian countries from 2018 [4,5]. Given that Asian countries account for ∼50% of the global pig production, Asia could serve as a reservoir of ASFV. The development of efficacious commercial vaccines against ASFV has been delayed because of limited information about the pathogenesis of ASFV infection, function of viral proteins, and host-virus interactions [6].

ASFV infection in pigs involves a complex interplay between the virus and host immune system. ASFV mainly infects macrophages and monocytes, as it enters the host through oral or nasal routes, causing them to migrate to the lymph nodes, where they trigger an adaptive immune response [7,8]. In the early stages of ASFV infection, Type I interferons (IFNs) and pro-inflammatory cytokines (*i.e.* tumour necrosis factor-α [*TNF-α*], interleukin [IL]−1β, and *IL-6*) are produced, which are crucial for preventing viral replication and distribution [8,9]. However, ASFV has developed a range of strategies to subvert and even escape the host’s innate antiviral defences, by inhibiting the production of Type I IFNs and suppressing dendritic and T cell activation, events that aid its multiplication and dissemination [10]. Several clinical studies have reported that ASFV initially spreads through the whole body *via* the blood or lymphatic fluid and then reaches various organs (*i.e.* the spleen, liver, lungs, and bone marrow) [11,12]. Whole-body disseminated ASFV triggers a systemic inflammatory response, releasing cytokines and chemokines that can lead to serious organ damage due to the cytokine storm syndrome [9,13]. However, limited studies have revealed the actual immune response in each organ of ASFV-infected pigs. Thus, there is a need to investigate the *in vivo* spread of ASFV antigens in pigs.

Most studies have conducted RNA sequencing analysis to reveal transcriptomic changes in porcine alveolar macrophages (PAMs) through *in vitro* experiments [9,13–15]. Significant alterations in gene expression can be observed in various organs (lungs, spleen, liver, kidneys, and lymph nodes) that are involved in innate immune response pathways, metabolic regulatory pathways, and inflammatory responses [12]. However, there is still a lack of studies on the transcriptome analysis of ASFV-infected pigs at the tissue level. Moreover, most previous studies have focused on analyzing changes in a single ASFV-infected organ at just one or two time-points, or multiple organs at one time-point [12,16,17]. This makes it essential to comprehensively elucidate the virus-host organ interactions and local transcriptomic responses from each organ during the early phase of infection, to understand the systemic molecular mechanisms of ASFV pathogenesis in pigs.

Here, we performed a time-series assessment of the viral spread in pigs experimentally inoculated (*via* the intravascular route) with ASFV (VNUA/HY/Vietnam), to determine the viral organ tropism for each tissue at an early stage of infection. In addition, the transcriptomes of various ASFV-infected organs were profiled one and three days after viremia. The outcomes of this study can contribute to our understanding of the underlying mechanisms driving the modulation of the host response in different organ tissues during the early phases of ASFV infection and facilitate the development of efficacious antiviral strategies in the future.

## Materials and methods

### Viral strain and experimental design

The viral strain used in this study was the first ASFV isolated in Vietnam (VNUA/HY/Vietnam, Embank accession no. MK554698). Virus isolation, propagation, and hemadsorption (HAD) assays were conducted according to our previous study [18]. HAD_50_/mL was calculated and a titre of 1 × 10^7^ HAD_50_/mL ASFV was prepared for inoculation of the experimental pigs.

A total of 52 healthy 6–7-week-old pigs (Yorkshire × Landrace) were obtained from a commercial pig farm in Hanoi, Vietnam. All pigs were seronegative for ASFV, porcine circovirus 2, foot-and-mouth disease virus, classical swine fever virus, and porcine reproductive and respiratory syndrome virus. The experimental pigs were allocated to the animal biosafety level 2 enhanced facility (ASBL-2+) at the National Institute of Veterinary Research, Hanoi, Vietnam, and randomly divided into two groups (TRT; *n *= 28 and negative control (NC); *n *= 24). Sixteen pigs (eight pigs per group) were euthanized before ASFV inoculation, and the remaining 20 pigs in the TRT group were individually intravascularly inoculated with 1 mL of 1 × 10^7^ HAD_50_/mL ASFV. Five pigs from the TRT group and four pigs from the NC group were then euthanized and necropsied, at different time-points [1, 2, 3, and 5 days post-inoculation (dpi)]. An overview of the study design is presented as a schematic representation (*SI Appendix*, Figure. S1).

### Sample collection and viral gene (p72) detection

The animals were clinically investigated daily; this included measurement of rectal temperature and evaluation of clinical signs of ASFV infection. Blood and three swab (oral, nasal, and rectal) samples were collected daily from the individual pigs until the pigs were euthanized, to evaluate viral loads by means of quantitative polymerase chain reaction (qPCR). The methods used for viral DNA extraction, qPCR, and reaction conditions have been described previously [18].

Necropsy was conducted immediately after euthanasia in all the experimental pigs (TRT, *n *= 28; NC, *n *= 24) sacrificed at 1, 2, 3, and 5 dpi. A total of nine tissue samples (approximately 1 g) consisting of spleen, submandibular lymph nodes (SLN), mesenteric lymph node (MLN), inguinal lymph node (ILN), tonsils, lungs, liver, kidneys, and heart were obtained from each necropsied pig and homogenized in 3 mL sterile phosphate-buffered saline. DNA was extracted from the homogenized tissues using a Qiagen DNA Mini Kit (Qiagen, Hilden, Germany). Viral loads were determined by means of qPCR, using the VDx® ASFV qPCR Kit (Median Diagnostics, Chuncheon, Korea).

### Organ tissue RNA extraction and sequencing, quality analysis, and differentially expressed gene (DEG) analysis

Eighteen necropsied pigs were randomly selected from the TRT and NC groups, at 0, 1, and 3 dpi (*n *= 3 per group and time-point). Total RNA was extracted from each homogenized tissue sample obtained from selected necropsied pigs using the Qiagen RNeasy Kit (Qiagen), according to the manufacturer’s instructions. RNA extraction and sequencing data processing, quality analysis, mapping of reads, and DEG analysis were performed as described previously [19].

### Functional analysis

To determine the biological significance and significant pathways associated with ASFV infection, DEGs, Gene Ontology (GO), and Kyoto Encyclopedia of Genes and Genomes (KEGG) pathway enrichment analyses were performed according to our previous study [19]. The genome of the pig (*Sus scrofa*) was chosen as the background parameter, and a threshold of *p *< 0.05 was chosen to denote a statistically significant difference.

### Protein – protein interaction (PPI) network construction

The PPI network of the 75 conserved DEGs in this study was analyzed according to a previous study [19]. We then selected the top three prime modules from the PPI network using the following cut-off criteria: node score cut-off = 0.2, degree cut-off = 2, maximum depth = 100, and k-core = 2.

### Statistical analyses

Statistical analyses of the time-series investigations of rectal temperature and viral load were performed using SPSS version 26.0 (IBM, Armonk, NY, USA). A linear mixed-effects model for repeated measures was used to assess the determinants of the time-course of rectal temperature. In this model, the experimental groups (TRT and NC) were included as fixed factors and time (dpi) as a random variable. The mean differences in rectal temperature were analyzed with respect to the group × time interaction. If this interaction was significant (*p *< 0.05), Student’s *t*-tests were used to compare the mean values of rectal temperature between the two groups of pigs at the same time-point. In addition, the mean viral loads from different organ tissues were analyzed using analysis of variance and Duncan’s test, to evaluate differences. The significance level was set at 5%. All data in this study are presented as mean ± standard deviation, calculated based on daily measurements in individual pigs.

## Results

### Clinical signs of ASF and ASFV viremia and excretion

Before viral inoculation, all 56 experimental pigs were healthy and presented a body temperature of 38.2–39.2°C. In the ASFV-infected group (TRT), at 2 dpi, one pig (6.3%, 1 of 16 pigs) showed high fever (>40.5°C) and at 3 dpi the average rectal temperature increased up to 40.8 ± 0.5°C (*SI Appendix*, Figure S2A). The mean temperatures gradually increased until 5 dpi (41.7 ± 0.1°C). As expected, the pigs in the NC group showed no high fever (<40.5°C) during the experimental period. There was a significant effect of time (dpi) and group × time interaction on the mean rectal temperatures of the TRT and NC groups (*p *< 0.001), with the average temperatures of the two groups differing significantly (*p *< 0.001) from following ASFV inoculation (1–5 dpi).

The average numbers of ASFV DNA copies in blood samples from TRT pigs gradually increased from 1 to 5 dpi (*SI Appendix*, Figure S2B); 1.0 × 10^6^ (1 dpi), 3.5 × 10^6^ (2 dpi), 4.2 × 10^6^ (3 dpi), 4.8 × 10^6^ (4 dpi), and 1.1 × 10^7^ copies/μL (5 dpi). All TRT pigs had viremia before euthanasia. At 1 dpi, ASFV DNA was detected in the nasal (1.3 × 10^1^) and rectal (9.5 × 10^0^ copies/μL) swab samples from one pig (6.3%; *SI Appendix*, Figure S2C), increasing to >50% of pigs at 2 dpi and all pigs at 4 dpi. In addition, the mean viral load from swab samples gradually increased until 5 dpi. At the last day of experiment (5 dpi), the average viral DNA copy number was the highest in nasal swab samples (1.3 × 10^5^), followed by oral (3.1 × 10^3^) and rectal (2.1 × 10^3^ copies/μL) swab samples.

### ASFV spreads to different organ tissues after viremia in a time-dependent manner

**Figure 1.**
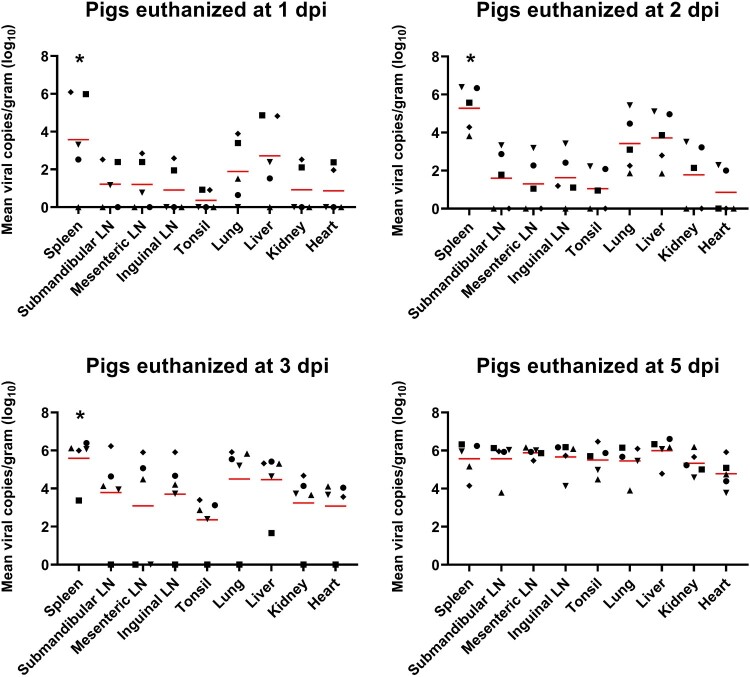
ASFV DNA copies in the various organ tissues (approximately 1 g) collected from pigs in the ASFV infection group. Red bars indicate the mean value in each tested organ tissue. *p < 0.05. LN: lymph node; ASFV, African swine fever virus; dpi, days post-infection

Figure 1 shows the time-dependent ASFV distribution in various organs of the TRT pigs. The mean number of viral copies per gram (log_10_) from pigs euthanized at 1, 2, and 3 dpi was significantly (1 dpi, *p *= 0.025; 2 dpi, *p *= 0.006; 3 dpi, *p *= 0.003) higher in the spleen than in the other organs, followed by liver, lungs, and SLNs at 1 dpi; liver, lungs, and kidneys at 2 dpi; and lungs, liver, and SLNs at 3 dpi. ASFV DNA was detected at a high viral load in all organ tissues collected at 5 dpi.

### Identification of DEGs among tissues in ASFV-infected pigs

Transcriptome analysis was performed on total RNAs from nine different organs (spleen, SLN, MLN, ILN, tonsils, lungs, liver, kidneys, and heart), at three time-points. We used DEGs without batch correction for principal component analysis to identify the underlying stratification in the samples, which revealed distinct clustering for each organ tissue and time-point (dpi) in the transcriptomes generated upon ASFV infection (*SI Appendix*, Figure S3).

Genes with low expression values (counts < 10) were removed, and DEGs between the TRT and NC pigs at 1 and 3 dpi were identified. *q *< 0.05 (adjusted *p*-value) and log_2_ fold-change (FC) ≥ 1.5 were set as the cut-off criteria for screening the DEGs. For pigs euthanized at 1 dpi, 293 (spleen), 4,903 (SLN), 5,635 (MLN), 165 (ILN), 5,679 (tonsils), 1,095 (lungs), 81 (liver), 667 (kidneys), and 339 (heart) DEGs were identified (*SI Appendix*, Figure S4A). Compared with NC pigs, TRT pigs euthanized at 3 dpi had 1,233 (spleen), 8,216 (SLN), 6,728 (MLN), 4,285 (ILN), 6,768 (tonsils), 5,506 (lungs), 1,346 (liver), 1,930 (kidneys), and 2,803 (heart) DEGs. A complete list of the DEGs in different tissue samples at 1 and 3 dpi is provided in *SI Appendix*, Datasets S1 and S2, respectively. At 1 and 3 dpi, most tissues from the TRT pigs showed similar dynamic changes in gene expression levels, with an increasing number of DEGs. There were more downregulated than upregulated genes in all ASFV-infected organ tissues at 1 dpi except for the MLN, ILN, and tonsils. However, at 3 dpi, there were fewer downregulated than upregulated genes in the lungs, kidneys, and heart. Hierarchical clustering analysis of the DEG expression patterns across multiple organ samples revealed that gene expression was significantly different at 0, 1, and 3 dpi (*SI Appendix*, Figure S4B). A heatmap for each tissue in the NC group at both the time-points was generated from normalized gene expression counts. *SI Appendix*, Figure S4B shows the expression patterns of the top 500 DEGs.

Volcano plots were generated (*SI Appendix*, Figure S5), and the top 5 DEGs were identified in various organ tissues from TRT pigs at 1 and 3 dpi (*SI Appendix*, Table S1). The overlapping upregulated and downregulated DEGs in the organ tissues are represented as a Venn diagram in *SI Appendix*, Figure S6. The list of genes found to be common between each organ tissue is shown in *SI Appendix*, Datasets S3. A total of 9,229 DEGs overlapped between pigs euthanized at 1 and 3 dpi, with 75 DEGs shared among all nine organs from TRT pigs euthanized at 1 and 3 dpi, respectively (Table 1).

**Table 1.** List of overlapping DEGs in the nine organ tissues obtained from ASFV-infected pigs euthanized at 1 and 3 dpi.

Group	Tissues	No. of genes	Overlapping DEGs
**3 dpi**	Spleen, SLN, MLN, ILN, tonsils, lungs, liver, kidneys, heart	74	*PRORP*, *DNAJB4*, *PPIDEIF4E*, *CYSLTR1*, *FCF1*, *BLZF1*, *DCHS1*, *NDUFA5*, *PSMA1*, *CHMP5*, *COL6A2*, *UBD*, *HSPH1*, *TSPAN6*, *PPA1*, *BIRC3*, *CAM*, *PPL*, *MIX23*, *FRS3*, *COL4A2*, *ENSSSCG00000031170*, *PLSCR1*, *HSPE1*, *RPS27L*, *SREBF1*, *AUTS2*, *PPARGC1B*, *KCP*, *TOM1L2*, *IFIT2*, *CDC42BPG*, *HR*, *GLI3*, *CLIC2*, *FUNDC1*, *FLRT1*, *DPY30*, *CHORDC1*, *TMEM156*, *UVSSA*, *CELSR2*, *LTBP3*, *ARL5B*, *KIAA1143*, *PRDX1*, *RBX1*, *MFSD1*, *PSMA2*, *RPL26*, *NT5C3A*, *FI44L*, *XD3*, *C21orf91*, *COL6A1*, *UBE2V2*, *COL5A1*, *CCT8*, *TMEM33*, *GABBR1*, *LSM3*, *ORMDL1*, *SNRPG*, *SOGA1*, *GPX8, DENND2B*, *HIST1*, *H2BD*, *SOX18*, *APLNR*, *DNAJA1*, *KBTBD11*, *IFIT5*
**1 dpi**	Spleen, SLN, MLN, ILN, tonsils, lungs, liver, kidneys, heart	8	*S100A8*, *IDO1*, *S100A12*, *ZSWIM9*, *CXCL10*, *TFEC*, *FRS3*, *SH2D1A*, *ARL5B*, *CXCL9*

dpi, days post-inoculation; SLN, submandibular lymph node; MLN, mesenteric lymph node; ILN, inguinal lymph node; ASFV, African swine fever virus; DEGs, differentially expressed genes

### Expression pattern of immune-related genes in the ASFV-infected pigs

**Figure 2.**
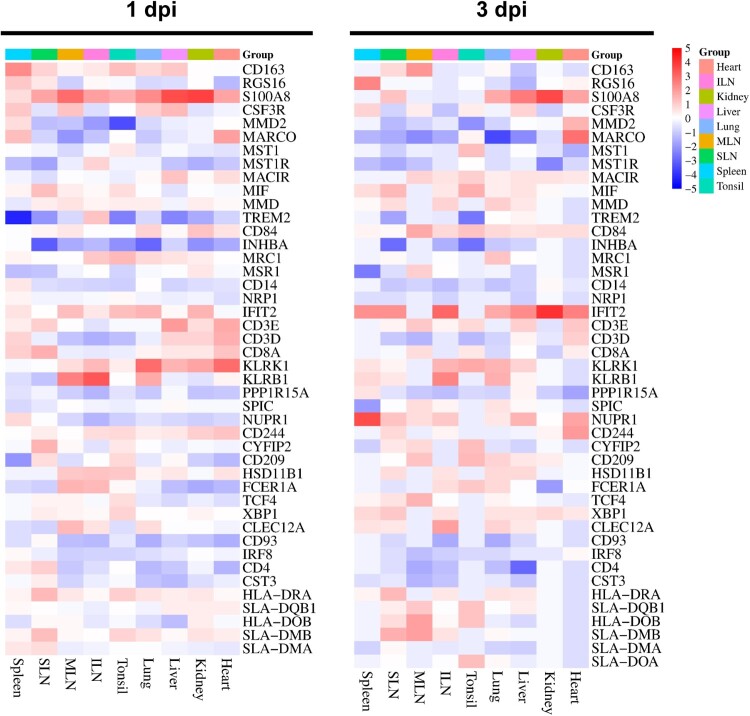
Heatmap plots representing the expression profile of DEGs involved in the different immune responses in each organ tissue from the ASFV-inoculated pigs (TRT pigs) euthanized at 1 and 3 dpi. SLN, submandibular lymph node; MLN, mesenteric lymph node; ILN, inguinal lymph node; DEGs, differentially expressed genes; ASFV, African swine fever virus

Based on recent studies, we investigated and highlighted viral infection or host defence immunity-related DEGs, including genes associated with macrophages, natural killer (NK) cells, T cells, other lymphocytes, class II major histocompatibility complex (MHC II), and ASFV infection [13,17]. The gene list, gene products, and their expression levels in different organs are shown in *SI Appendix*, Datasets S4 and the expression levels of these genes in the organs and tissues of TRT pigs euthanized at 1 and 3 dpi are shown in Figure 2.

In TRT pigs euthanized at 1 dpi, the macrophage-related genes, including *CSF3R* (MLN: 3.34-fold) and *S100A8* (MLN: 6.75-fold, kidneys: 5.23-fold, SLN: 3.40-fold, liver: 3.09-fold, and tonsils: 3.08-fold), displayed elevated expression levels; NK cell-associated genes *IFIT2* (MLN: 3.59-fold), *KLRB1* (MLN: 5.90-fold), and *KLRK1* (lungs: 3.26-fold and heart: 3.65-fold) were upregulated by >3 – to 5-fold; lymphocyte-related genes (*CLEC12A*, *FCER1A*, and *HSD11B1*) were substantially expressed in the MLN (3.63-, 3.94-, and 3.00-fold, respectively); and the T cell-related gene, *CD8A*, was significantly expressed in the SLN (3.08-fold).

At 3 dpi, the macrophage-associated genes, *CD163* (SLN: 3.18-fold), *MARCO* (heart: 4.55-fold), *MIF* (SLN: 4.37-fold), *RSG16* (spleen: 4.12-fold), *S100A8* (heart: 3.13-fold, kidneys: 4.04-fold, SLN: 4.01-fold, liver: 3.67-fold, lungs: 3.46-fold, and spleen: 4.12-fold), were significantly upregulated (>3-fold); NK cell-associated genes, *IFIT2* (SLN: 5.93-fold, ILN: 4.89-fold, kidneys: 4.40-fold, heart: 4.05-fold, spleen: 3.76-fold, lungs: 3.44-fold, and liver: 3.18-fold), *KLRB1* (ILN: 4.06-fold and lungs: 3.38-fold), and *KLRK1* (lungs: 3.22-fold), were upregulated by >3-fold; expression of the ASFV-related gene, *NUPR1*, was elevated by more than 5.93-fold in the spleen, 3.88-fold in the SLN, and 3.21-fold in the heart; two lymphocyte-related genes showed upregulated expression, *CLEC12A* (3.34-fold) in the ILN and *XBP1* (3.69-fold) in the SLN; and MHC-related genes (*HLA-DRA* and *SLA-DMB*) were substantially upregulated (4.37 – and 4.68-fold, respectively) in the SLN.

### Expression pattern of cytokines in ASFV-infected pigs

The expression levels of various IFNs, ILs, the TNF superfamily, chemokines, and their receptors were evaluated in each organ tissue from the TRT pigs at 1 and 3 dpi (*SI Appendix*, Datasets S4). In TRT pigs euthanized at 1 dpi, various chemokines and their receptors (*CCL16*, *CCL2*, *CCL26*, *CCR1*, *CCR4*, *CCR5*, *CCR9*, *CCRL2*, *CXCL10*, *CXCL11*, *CXCL8*, *CXCL9*, *CXCR4*, and *CXCR6*) were highly expressed (>3-fold) in the heart, ILN, kidneys, lungs, MLN, SLN, spleen, and tonsils; a total of 21 IFNs, ILs, and allied genes (*IFI44*, *IFI44L*, *IFI6*, *IFIH1*, *IFIT1*, *IFIT2*, *IFIT5*, *IFN-DELTA-1*, *IFNG*, *IFNGR1*, *IL12A*, *IL13RA1*, *IL13RA2*, *IL1R2*, *IL21*, *IL22RA2*, *IL2RG*, *IL33*, *IL7R*, *ILF2*, and *ILF3*) were upregulated (>3-fold) in various tissues (heart, kidneys, liver, MLN, SLN, and tonsils); and the TNF superfamily and associated genes, such as *TNFAIP6*, *TNFRSF17*, *TNFRSF9*, *TNFSF13B*, and *TNFSF18*, were highly expressed (>3-fold) in the liver, MLN, and tonsils.

**Figure 3.**
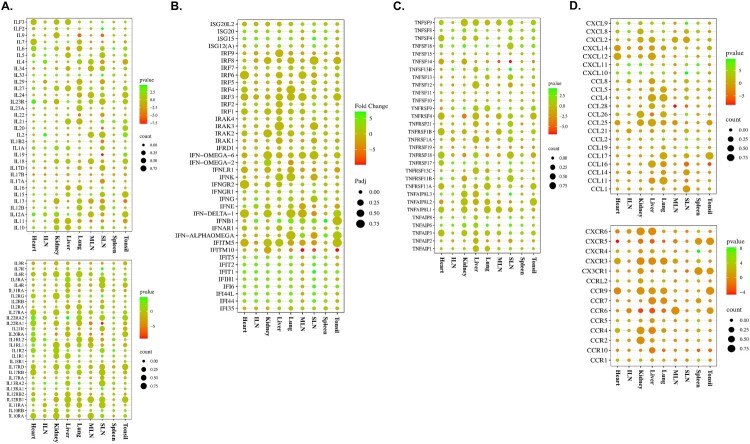
Gene expression patterns of cytokines and their receptors in organ tissues (tonsils, spleen, SLN, MLN, lungs, liver, kidneys, ILN, and heart) from ASFV-inoculated pigs (TRT) euthanized at 3 dpi. **(A)** ILs and their receptors. **(B)** IFNs. **(C)** Genes of the TNF-receptor superfamily. **(D)** Chemokines and their receptors. SLN, submandibular lymph node; MLN, mesenteric lymph node; ILN, inguinal lymph node; TNF, tumour necrosis factor; ASFV, African swine fever virus; IFN, interferon; IL, interleukin

Figure 3 shows the expression patterns of cytokines and their receptors in TRT pigs euthanized at 3 dpi: ILs (*IL12A*, *IL2*, *IL23R*, *IL33*, *IL4*, *IL5*, and *IL6*) and their receptors (*IL13RA1*, *IL13RA2*, *IL1R2*, *IL22RA2*, *IL2RG*, and *IL7R*) were upregulated (>3-fold) in the heart, ILN, liver, lung, spleen, and SLN tissue samples (Figure 3(a)); a total 15 IFNs and associated genes [*IFI44L*, *IFI6*, *IFIT1*, *IFIT2*, *IFIT5*, *ISG15*, *ISG20*, *IFI44*, *IFIH1*, *IFNB1*, *IFNG*, *ISG12(A)*, *IFN-ALPHAOMEGA*, *IFNGR1*, and *IRF2*] were upregulated (>3-fold) in a variety of tissues, including the tonsils, heart, ILN, kidneys, liver, and MLN (Figure 3(b)); various organs (heart, ILN, kidneys, lungs, spleen, and SLN) showed significantly upregulated levels (>3-fold) of the TNF-receptor superfamily genes, including *TNFAIP6*, *TNFAIP8*, *TNFAIP8L3*, *TNFRSF11B*, *TNFRSF17*, *TNFSF13B*, and *TNFSF18* (Figure 3(c)); and the chemokines *CCL19*, *CCL2*, *CCL26*, *CXCL10*, *CXCL11*, *CXCL12*, *CXCL2*, *CXCL8*, and *CXCL9* (heart, ILN, kidneys, liver, lungs, spleen, and SLN), and their receptors *CCR1*, *CCR2*, *CCR4*, *CCR5*, *CCRL2*, *CXCR4*, and *CXCR6* (heart, spleen, and SLN) were expressed >3-fold in several tissues (Figure 3(d)). *SI Appendix*, Datasets S4 contains information on the expression levels of all the cytokine genes and their corresponding host organ tissues. The heart, spleen, and SLN displayed the highest level of cytokine expression among all the ASFV-infected organ tissues.

### Functional and pathway enrichment analyses of the DEGs

To gain a better understanding of their biological processes and pathway enrichment in the pigs’ transcriptomic responses to ASFV infection, GO and KEGG pathway enrichment analyses were performed using DEGs that were significantly (FC > 1.5-fold and p_adj _< 0.05) upregulated and downregulated in all the organ tissues from TRT pigs euthanized at 1 and 3 dpi. All relevant GO biological processes (BP) and KEGG pathways were separately collected from each organ tissue during the early ASFV infection period (1 and 3 dpi). The enrichment of the total pathways is shown as a percentage for each individual organ tissue, at both the time-points (*SI Appendix*, Figure S7A). The KEGG analysis results revealed that the DEGs were successfully enriched in a total of 7 (spleen), 77 (SLN), 85 (MLN), 6 (ILN), 116 (tonsils), 46 (lungs), 4 (liver), 23 (kidneys), and 10 (heart) pathways (*p *< 0.05) at 1 dpi (*SI Appendix*, Figure S7B) and 62 (spleen), 72 (SLN), 70 (MLN), 75 (ILN), 93 (tonsils), 96 (lungs), 41 (liver), 49 (kidneys), and 95 (heart) pathways (*p *< 0.05) at 3 dpi. In addition, an UpSetR plot was created to provide a better assessment of the number of distinct and shared pathways between organ tissues from pigs euthanized at 1 and 3 dpi (*SI Appendix*, Figures S7C and S7D, respectively).

Notably, a large number of pathways were significantly enriched in every organ tissue from ASFV-infected pigs euthanized at 1 and 3 dpi (*SI Appendix*, Datasets S5, Table S2 and Table S3). Genes related to metabolic KEGG pathways were most frequently detected in the SLN, MLN, and tonsils of TRT pigs, at 1 and 3 dpi. However, these were most prevalent in the ILN, lungs, and liver at 3 dpi, but not at 1 dpi.

Further analysis was conducted to focus on the major immune-related pathways shared by two or more organ tissues, to obtain a better understanding of the host responses against ASFV infection. Of the total pathways, only nine conserved immune-related pathways, including the phosphoinositide 3-kinases (PI3K)-Akt signalling pathway, IL-17 signalling pathway, viral protein interaction with cytokines and cytokine receptors, primary immunodeficiency, T cell receptor signalling pathway, viral carcinogenesis, complement and coagulation cascades, TGF-β signalling pathway, and cytokine-cytokine receptor interaction, were significantly (*p *< 0.05) enriched at 1 dpi (Table 2). A total of 10 shared immune-related pathways were observed at 3 dpi, namely the PI3K-Akt signalling pathway, nuclear factor-kappa B (NF-κB) signalling pathway, chemokine signalling pathway, IL-17 signalling pathway, complement and coagulation cascades, Toll-like receptor signalling pathway, T cell receptor signalling pathway, cytokine-cytokine receptor interaction, TGF-β signalling pathway, and viral protein interaction with cytokine and cytokine receptor. Notably, the PI3K-Akt signalling pathway was concomitantly detected in all organs at both time-points (1 and 3 dpi). Another immune-associated pathway, the NF-κB signalling pathway, was shared in every organ at 3-dpi.

**Table 2.** List of overlapping immune-associated pathways that were significantly (*p* < 0.05) enriched with DEGs identified in organ tissues from ASFV-infected pigs, at 1 and 3 dpi

Group	Overlapping immune-related pathways	Tissues	Top overlapping genes
**1 dpi**	PI3K-Akt signalling pathway	Heart, ILN, kidney, liver, lung, MLN, muscle, spleen, SLN, tonsils	*COL4A2*, *COL6A2*, *COL6A1*, *NGFR*, *COL9A3*
	IL-17 signalling pathway	ILN, kidneys, liver, MLN, tonsils, spleen	*S100A8*, *IL5*, *CXCL10*, *LCN2*, *HSP90AA1*
	Viral protein interaction with cytokine and cytokine receptor	Kidneys, lungs	*CCR10*, *CXCL9*, *CXCL10*, *CCR1*, *IL22RA1*
	Primary immunodeficiency	Heart, kidneys, SLN	*ICOS*, *CD8A*, *LCK*, *CD3E*, *PTPRC*
	T cell receptor signalling pathway	Heart, kidneys, MLN	*ICOS*, *ITK*, *CD3E*, *MAPK11*, *IL5*
	Viral carcinogenesis	MLN, muscle, SLN	*IL6ST*, *HPN*, *PRKACB*, *SNW1*, *NRAS*
	Complement and coagulation cascades	Heart, ILN, kidneys, lungs, tonsils	*C6*, *C7*, *FGB*, *TFPI*, *CFI*
	TGF-β signalling pathway	MLN, SLN, tonsils	*INHBA*, *SP1*, *DCN*, *PPP2R1B*, *SMURF2*
	Cytokine-cytokine receptor interaction	Heart, ILN, kidneys, lungs	*CXCL9*, *CXCL10*, *IL33, ACVR1C*, *BMP10*
**3 dpi**	PI3K-Akt signalling pathway	Heart, ILN, kidneys, liver, lungs, MLN, muscle, spleen, SLN, tonsils	*COL4A2*, *COL6A2*, *COL4A1*, *LAMA5*, *COL1A1*
	NF-κB signalling pathway	Heart, ILN, kidneys, liver, lungs, MLN, muscle, spleen, SLN, tonsils	*CD40*, *VCAM1*, *CCL4*, *IRAK4*, *IL1R1*
	Chemokine signalling pathway	Heart, kidneys, liver, lungs, MLN, muscle, SLN, tonsils, spleen	*CXCL11*, *CXCL9*, *CXCL10*, *CCL2*, *CCL19*
	IL-17 signalling pathway	Heart, ILN, kidneys, liver, lungs, MLN, tonsils, spleen	*CXCL10*, *CCL2*, *MAPK7*, *CXCL8*, *HSP90B1*
	Complement and coagulation cascades	Heart, kidneys, liver, lungs, SLN, tonsils, spleen	*C1QB*, *C5*, *C7*, *C1QA*, *C1QC*
	Toll-like receptor signalling pathway	Heart, ILN, kidneys, spleen	*CXCL10*, *LY96*, *CXCL9*, *CXCL11*, *CCL4*
	T cell receptor signalling pathway	Heart, ILN, muscle, spleen	*NRAS*, *CD3E*, *JUN*, *ITK*, *PPP3CA*
	Cytokine-cytokine receptor interaction	Heart, kidneys, liver, spleen	*CXCL10*, *CCL2*, *CXCL11*, *CXCL9*, *IL17B*
	TGF-β signalling pathway	Heart, lungs, SLN, tonsils	*TGFBR1*, *PPP2R1B*, *NOG*, *RBX1*, *ZFYVE16*
	Viral protein interaction with cytokine and cytokine receptor	Heart, liver, spleen	*CXCL8*, *CXCL10*, *CXCL9*, *CCL2*, *CXCL11*

SLN, submandibular lymph node; MLN, mesenteric lymph node; ILN, inguinal lymph node; NF-κB, nuclear factor-kappa B; TGF-β, transforming growth factor-beta; PI3 K, phosphoinositide 3-kinase; IL, interleukin

**Figure 4.**
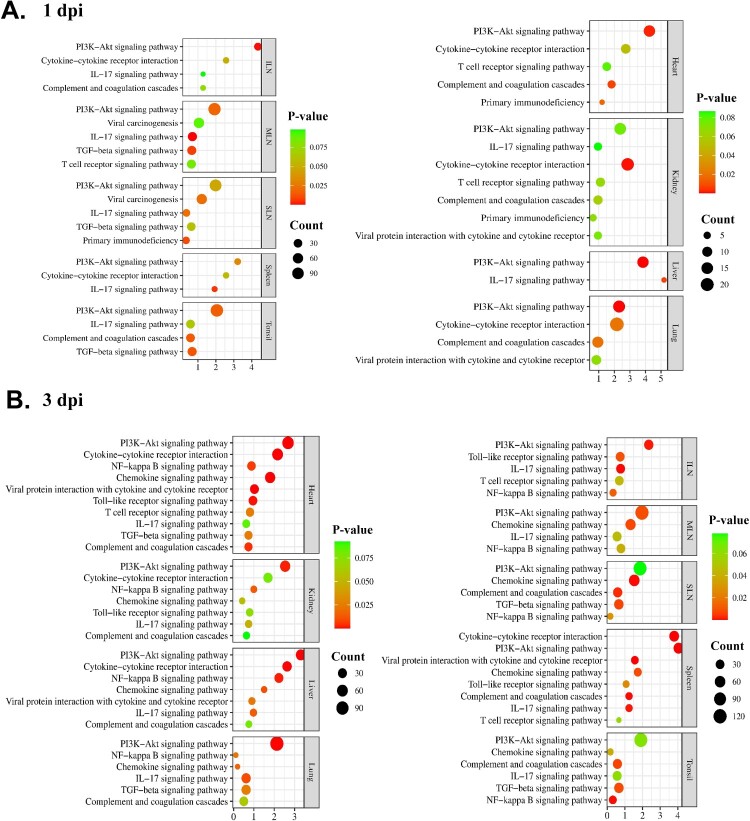
Illustration of the conserved immune-related pathways in organ tissues (the spleen, SLN, MLN, ILN, tonsils, lungs, liver, kidneys, and heart) from ASFV-inoculated pigs (TRT) euthanized at **(A)** 1 and **(B)** 3 dpi. The KEGG pathways terms are listed on the left, and *p*-value and gene counts are shown on the right. SLN, submandibular lymph node; MLN, mesenteric lymph node; ILN, inguinal lymph node; KEGG, Kyoto Encyclopedia and Gene and Genomes; ASFV, African swine fever virus; IL, interleukin; PI3 K, phosphoinositide 3-kinase; NF-κB, nuclear factor-kappa B; dpi, days post-inoculation

GO-BP functional enrichment analysis showed that 32 (spleen), 134 (SLN), 112 (MLN), 17 (ILN), 127 (tonsils), 86 (lungs), 23 (liver), 51 (kidneys), and 23 (heart) GO terms were significantly (*p *< 0.05) enriched at 1 dpi and 101 (spleen), 148 (SLN), 146 (MLN), 100 (ILN), 149 (tonsils), 108 (lungs), 92 (liver), 85 (kidneys), and 154 (heart) GO terms were significantly (*p *< 0.05) enriched at 3 dpi (*SI Appendix*, Datasets S4 and S5). Figure 4(a,b) represent the conserved immune-related pathways of each organ tissue from TRT pigs, at 1 and 3 dpi, respectively, and their gene counts with *p*-value.

### PPI network, module analysis, and hub gene selection

**Figure 5.**
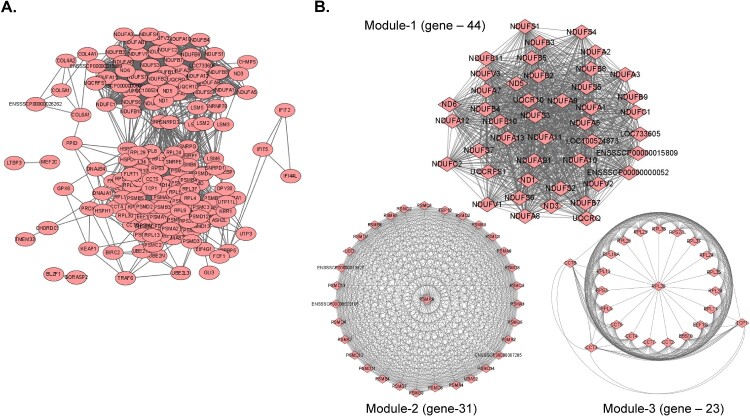
**(A)** PPI network for the 75 common DEGs identified in the nine organ tissues (spleen, submandibular lymph node, mesenteric lymph node, inguinal lymph node, tonsils, lungs, liver, kidneys, and heart) from ASFV-inoculated pigs (TRT pigs) euthanized at 3 dpi. A total 164 nodes and 2,444 interaction associations were detected. **(B)** Module analysis from the PPI network. The Top 3 significant modules (Module 1, 2, and 3) were obtained from the PPI network of DEGs, using MCODE. PPI, protein-protein interaction; ASFV, African swine fever virus; DEGs, differentially expressed genes; dpi, days post-inoculation.

A PPI network was constructed with the 75 conserved DEGs observed at 3 dpi in every organ tissue using the STRING database, with 164 nodes and 2,444 edges (Figure 5(a)). At a degree cut-off of 10, 160 genes were identified in the PPI network. The top 10 hub genes with the highest degrees to the other nodes were *UBA52*, *RPS3*, *PSMA4*, *PSMA3*, *BBS10*, *UBD*, *PSMA7*, *PSMA8*, *NDUFA1*, and *CCT5* (*SI Appendix*, Table S4).

Furthermore, the MCODE plugin was used to evaluate the entire PPI network, to identify key PPI network modules and simplify the comprehension of the molecular mechanisms. The PPI network yielded a total of seven modules, with a node score cut-off of 0.2 and degree cut-off of 2. Tissues from TRT pigs at 1 dpi showed a relatively low number of shared genes (eight genes), thus making it difficult to construct a PPI network. Therefore, PPI network analysis was performed only for organ tissues from TRT pigs at 3 dpi (*SI Appendix*, Datasets S6). The top 3 important clusters with a k-core > 6 were designated as sub-networks and named modules 1, 2, and 3 (Figure 5(b)). The genes in module 1 were strongly associated with metabolic pathways, oxidative phosphorylation, diabetic cardiomyopathy, chemical carcinogenesis, tumorigenesis, retrograde endocannabinoid signalling, Parkinson’s disease, prion disease, non-alcoholic fatty liver disease, Huntington’s disease, amyotrophic lateral sclerosis, Alzheimer’s disease, neurodegeneration, metabolic pathways, and cardiac muscle contraction pathways. Module 2 showed strong associations with the proteasome, spinocerebellar ataxia, Parkinson’s disease, prion disease, Huntington’s disease, amyotrophic lateral sclerosis, pathways of neurodegeneration, multiple diseases, Alzheimer’s disease, and Epstein – Barr virus infection pathways. Ribosome and coronavirus disease-2019 (COVID-19) were the key pathways associated with the genes in Module 3.

## Discussion

The outbreak of ASF has inflicted substantial economic losses on the global swine industry [12,13]. Despite numerous attempts by researchers to develop vaccines against ASFV, none are currently commercially available [20-23]. This can be attributed to the limited understanding of the mechanisms underlying ASFV-host interactions and their pathogenesis [13,23,24]. Recently, numerous *in vitro* studies conducted comprehensive transcriptome profiling of ASFV-infected PAMs to unravel virus-host interactions [13-15]. However, to establish efficient antiviral strategies, it is essential to understand how the organs of ASFV-infected pig function and are regulated against ASFV. The lack of information on host dynamic transcriptome changes in different organs after ASFV infection in pigs has limited our understanding of the pathobiological process. In this study, we revealed the complex interplay between ASFV organ tissue tropism during the early phase of infection in pigs, host organ responses at the transcriptome level, and the immune mechanisms to counteract viral infection. The outcomes of this study provide a better understanding of the functional attributes of individual organ tissues in ASFV-infected pigs and elucidate the critical signalling pathways and DEGs associated with innate immunity modulation triggered by viral infection.

Our previous study demonstrated that the ASFV (VNUA/HY/Vietnam)-inoculated pigs started to show viremia at from 1 to 4 dpi (average 2.2 ± 0.8 dpi), implying that individual differences exist when the pigs were intramuscularly inoculated with the virus [18]. In this study, all experimental pigs were intravascularly inoculated with a high dose of ASFV (1 mL of 1 × 10^7^ HAD_50_/mL), to synchronize the timing of the ASFV infection. Although this inoculation method could not reflect the natural infection status of ASFV, it was possible to analyze the organ tissue tropism of ASFV and transcriptomic responses of each organ tissue after viremia as time series. The TRT group displayed a significantly (*p *< 0.001) higher rectal temperature than the NC group, from 1 to 5 dpi, and showed high fever (>40.5°C) at 3 dpi.

We also investigated viral DNA copies in blood, oral swab, nasal swab, and rectal swab samples from 0 to 5 dpi, to confirm that all TRT pigs were successfully infected with ASFV. Using this infection model, we revealed the time-dependent viral tissue tropism of ASFV in multiple organs of pigs during the early phase of viremia (1, 2, 3, and 5 dpi). The average ASFV DNA copy numbers in the spleens of TRT pigs were significantly (*p *< 0.05) higher than those in the other eight organs (SLN, MLN, ILN, tonsils, lungs, liver, kidneys, and heart) at 1, 2, and 3 dpi, although individual variations were observed. This result agrees with previous studies that showed that the spleen and lymph nodes had the highest viral content in ASFV-infected pigs [12,24]. However, the results of this study demonstrated that the three lymph nodes (SLN, MLN, and ILN) showed relatively lower viral loads than the lungs and liver at 1, 2, and 3 dpi. In addition, all organ tissues from TRT pigs euthanized at 5 dpi showed a high average number of ASFV DNA copies. Altogether, our data indicated that ASFV spread rapidly to the spleen after viremia and was broadly transmitted to the liver and lungs, followed by the SLN, ILN, kidneys, heart, MLN, and tonsils.

However, it should be noted that our study had a limitation in that it did not accurately recapitulate natural ASFV infection in pigs. In this study, all TRT pigs were intravascularly inoculated with high dose of ASFV for synchronizing viremia; however, the main routes of ASFV infection are ingestion of virus contaminated feed or contact with infected pigs [25]. The swallowed ASFV particles initially replicate in macrophages in the tonsils or SLNs and then spread to whole body through the blood and lymphatic vessels [26]. Moreover, given that the spleen and liver have a relatively large blood volume compared to other organs, the amount of blood could be one of the reasons for the high viral copy numbers in these organs at the initial period of ASFV intravenous inoculation. Nevertheless, the time-serial transcriptome in ASFV-infected organ tissues should be evaluated without individual variation in onset timing of viremia. Therefore, we further analyzed the gene expression patterns during the early phase of viremia (1 and 3 dpi) in each organ tissue of pigs experimentally inoculated with ASFV.

A clearly delineated trajectory of the transcriptomes associated with ASFV infection was detected in individual tissues, using principal component analysis. The number of DEGs identified in each organ differed with the time after viremia, because the primary target immune cells of ASFV (monocytes-macrophages) are different in composition across organ tissues. Therefore, the release of effector molecules by ASFV-infected cells afterward depended on the organ and infection time (dpi), leading to variable outcomes [12,27]. This could explain why the spleen and liver exhibited a lower number of DEGs than all other organs, despite maintaining the highest number of DNA viral copies at 1 and 3 dpi. Furthermore, the dramatic increase in the number of DEGs at 3 dpi in these organs may be due to the severity of the infection and the host immune response.

Previous studies have suggested that ASFV infection in pigs triggers a variety of immune mechanisms, including activation of the monocyte/macrophage lineage, to regulate the release of inflammatory cytokines, apoptosis of CD4^+^ and CD8^+^ T and NK cells, and suppression of M1 activation of macrophages [10,15,28]. In this study, we investigated the expression levels of genes associated with immune cells and their antiviral activities across multiple organs during ASFV infection, at both 1 and 3 dpi. The expression of macrophage-related genes (*CD163*, *MARCO*, *MIF*, *S100A8*, and *CLEC12A*) increased notably (>3-fold) at 3 dpi, along with that of *CSF3R* and *S100A8* at 1 dpi.

These genes have been reported to play key roles in inflammatory processes and the innate immune system during ASFV pathogenesis [13,28-30], which correlates with a comprehensive host organ immune response by activating lymphocytes and myeloid cells. Interestingly, *S100A8* exhibited substantial upregulation (>3-fold) across multiple organs at 1 and 3 dpi. The crucial functions of *S100A8* consist of influencing the inflammatory response, stimulate leukocyte recruitment, and trigger cytokine secretion [28]. A recent study also showed that *S100A8* was only upregulated in blood samples from pigs infected with highly pathogenic strains (Georgia 2007/1) and not in those infected with a low-pathogenic strain (OURT 88/3) [16]. Our *in vivo* experiment revealed that this host gene (*S100A8*) could play an important role in determining ASFV severity and intensifying macrophage secretory activity and the release of inflammatory cytokines in the early stage of ASFV infection.

In contrast, three NK cell-associated genes (*IFIT2*, *KLRB1*, and *KLRK1*) were upregulated (>3-fold) in multiple organs at 1 and 3 dpi. The T cell-specific *CD8A* was upregulated at 1 dpi in different organs, especially in the lymph nodes, indicating the antiviral response of the host immune system by activating NK cells before the development of the adaptive immune response [17,31]. The Fc epsilon receptor expressed in dendritic cells, *FCER1A*, was upregulated in the lymph nodes at 1 dpi, implying that pathogen sensing, phagocytosis, and antigen presentation occurred in the lymph nodes in the early phase of infection [32].

The lower expression levels of MHC II-associated genes (*SLA-DRB1*, *HLA-DRA*, *SLA-DQB1*, *HLA-DOB*, *SLA-DMB*, *SLA-DMA*, and *SLA-DOA*) at 1 and 3 dpi can be explained by the fact that ASFV infection suppresses MHC II antigen processing and presentation after macrophage infection [15,17]. Furthermore, significant upregulation of the autophagy-associated gene *NUPR1* was observed in the spleen, SLN, and heart at 3 dpi but no notable expression was observed at 1 dpi. Given that elevated *NUPR1* expression leads to deactivation of the autophagy-associated apoptosis induced by metabolic stress [13,15,33], our results suggest that ASFV affects multiple organs (spleen and lymph nodes) to express *NUPR1* at an early stage of infection, for survival and replication.

ASFV pathogenesis involves a significant contribution from an intense pro-inflammatory cytokine response often referred to as a “cytokine storm” [15,34]. Likewise, our findings suggested a potential fundamental role of chemokines in ASF pathogenesis in the heart, ILN, kidneys, liver, lungs, spleen, and SLN at 1 and 3 dpi, as evidenced by the notably heightened upregulated expression of nine chemokines (*CCL2*, *CCL19*, *CCL26*, *CCL16*, *CXCL2*, *CXCL10*, *CXCL11*, *CXCL8*, and *CXCL9*), which have key roles in monocyte, CD4^+^ T, CD8^+^ effector T, Th1 type T, Th2 type T, and NK cell activation and recruiting [15,17,35]. Although a previous *in vitro* study suggested that there was decreased expression of *CXCL8* in the PAMs after ASFV inoculation [9], we observed induction of the neutrophil chemoattractant chemokines *CXCL8* and *CXCL2* in our *in vivo* study. Additionally, our findings agree with a recent study demonstrating *CXCL10* stimulation in the spleen following ASFV infection [17]. Machuka *et al.* [17] also proposed that this stimulation may drive lymphocyte activation toward the Th1 phenotype or initiate apoptosis in T lymphocytes.

Significant upregulation in the expression of ILs and IFNs was observed at 1 and 3 dpi, which can be attributed to an early defence mechanism against ASFV infections during the primary immune response of cells across multiple organs. IFN-stimulated genes, including *IFIT1*, *IFIT2*, *IFIT5*, *IF16*, and *IFIH1*, were upregulated in organ tissues at 1 and 3 dpi. These genes are generally upregulated during ASFV infection [9]. *IFIH1* functions as an intracellular sensor of viral RNA, initiating the innate immune response, and subsequently triggering a pro-inflammatory reaction that involves the production of IFNs [36]. We also identified the prominently expressed IFN-stimulated genes *ISG15* and *ISG20*. The induction of *ISG15* and *ISG20* in PAMs during the initial phase of ASFV infection in various strains has been reported previously [13,15,37]. The role of *IFNG* (*IFN-γ*), one of the main IFNs, in ASFV infection remains controversial. *IFNG*-dependent activation of innate immunity against ASFV represents essential immune mechanisms contributing to the defence against ASFV [38,39], whereas studies have also reported that upregulated levels of *IFNG* expression are not necessarily associated with the protection of domestic pigs [38,40]. In the present study, substantial levels of *IFNG* expression were detected in multiple organs at 1 and 3 dpi. Among all ILs, *IL-33* and *IL-6* were the top two upregulated cytokines at both the time-points, which is similar to previous studies on ASFV-infected PAMs and organ tissues [9,17]. The pro-inflammatory cytokine, *IL-33*, is constitutively expressed in healthy tissues and serves as an “alarmin” that signals cell or tissue damage to immune cells [37,41]. *IL-6* is a multifunctional cytokine with pro-inflammatory and anti-inflammatory properties [42]. In addition, *IL-6* triggers pro-inflammatory effects, such as monocyte recruitment to the site of inflammation due to viral infection [38]. Furthermore, other pro-inflammatory cytokines (*IL-2* and *IL-4*) were upregulated in the heart, ILN, liver, lungs, spleen, and SLN at 3 dpi; these increases may be due to the enhancement of T and NK cell cytotoxicity during ASFV infection [38,43,44]. In agreement with previous studies [15,42], *IL-18* (belonging to the IL-1 superfamily) was downregulated at both the time-points, which may be due to the suppression of *IL-18* signalling by ASFV infection [15,45]. The pro-inflammatory TNF superfamily ligands *TNFSF13B* and *TNFSF18* were upregulated at 1 and 3 dpi, which agrees with the results of a recent transcriptome study on PAMs [15]. *TNFSF13B* contributes to the activation, proliferation, and differentiation of B lymphocytes [45], while *TNFSF18* regulates the activation of monocytes and T lymphocytes in the immune response against pathogen infection [46]. Our results suggest that excessive production of these two TNF cytokines in multiple organs during the early stages of ASFV infection could be a major factor in ASFV pathogenesis and severity.

Intracellular metabolic reprogramming is a well-known pathway that supports viral replication in multiple viral infections, including ASFV [47]. One interesting finding of the current study was that the metabolic KEGG pathway was most frequently observed in the tonsils, SLN, and MLN at 1 dpi, suggesting that these organs were affected by ASFV in the very early stage of viremia, to facilitate viral replication. Previously, ASFV has been shown to first replicate in monocytes/macrophages from lymphatic organs close to the initial site of infection; therefore, the tonsils and SLN are the major initial replication sites in pigs naturally infected with ASFV *via* the oronasal route [26]. However, our findings present a novel perspective that the tonsils, SLN, and MLN are major organs for viral replication in the early stages of viremia, regardless of the ASFV infection route. At 3 dpi, the number of DEGs involved in metabolic pathways was still the highest in the tonsils, ILN, and MLN. Notably, metabolic KEGG pathways were the most common pathways in the ILN, lungs, and liver, at 3 dpi. These results suggest both PAMs and Kupffer cells contribute to the host response that facilitates ASFV replication at 3 d after viremia.

Among the immune-related KEGG pathways, the PI3K-Akt signalling pathway was concomitantly detected in all organ tissues at 1 and 3 dpi. Previous studies have demonstrated that various viruses use different strategies to exploit the PI3K-Akt signalling pathway, to extend their replication in acute and persistent infections [48,49]. Moreover, activation of the PI3K-Akt signalling pathway shortly after viral infection can result in immediate cellular responses to enhance viral entry and sustained reactions with long-term effects [49]. Interestingly, our results indicated that collagen-associated genes were enriched in the PI3K- Akt signalling, in all ASFV-infected organs, at both 1 and 3 dpi. Given that these collagen genes function in actin cytoskeleton rearrangements [50], they may facilitate a macropinocytosis-driven endocytic process for ASFV entry in the early phase of infection [48].

One interesting finding of the current study is that NF-κB signalling pathway-associated genes were found to be strongly upregulated in all organs at 3 dpi, but in none at 1 dpi. The NF-κB signalling pathway plays a crucial role as a signalling cascade to activate innate defence pathways during virus infection, thereby regulating the transcription of antiviral genes like IFNs, cytokines, and chemokines, as well as modulators, immunoreceptors, cell cycle regulators, and genes associated with apoptosis and stress response [51]. In addition, this pathway is reported to trigger ASFV infection, and its inhibition has the potential to impede viral replication [52]. For this reason, the NF-κB signalling pathway has received extensive attention as a potential immunomodulatory pathway against ASFV infection [52,53]. Recently, numerous studies suggested through *in vitro* experiments that ASFV activated the NF-κB signalling pathway at the early phase of infection (appropriately 3–6 h after viral inoculation) [13,26,52]. Additionally, a recent single cell RNA sequencing study revealed that the TNFA signalling pathway via NF-κB and p53 pathway were activated in spleen macrophages following ASFV infection, leading to massive macrophage death [54]. However, our *in vivo* experiments showed that the pathway was not activated at a very early time-point after viremia (1 dpi) but was pronounced in all organs (except the spleen) at 3 dpi. These findings provide a new insight that the PI3K-Akt signalling pathway may play a more significant role than the NF-κB signalling pathway during the very early stage of ASFV infection (viremia) in infected pigs. There is a need for future studies that focus on these major pathways, as a better understanding of them could facilitate the development of efficacious antiviral strategies. Moreover, a recent study using single cell RNA sequencing indicated that the transition of ASFV infection from macrophages to immature monocytes was a critical factor for the persistence of viral infection [54]. Therefore, further studies involving single cell transcription profiles of ASFV-infected cells from various organ tissues could provide valuable insights into the pathogenesis of ASFV infection.

A PPI network was constructed to gather valuable interaction information regarding commonly enriched DEGs across the nine tissues at 3 dpi. In this study, *UBA52* was ranked as the top hub gene and was upregulated (>1 fold), as compared to all other hub genes. *UBA52* encodes a fusion protein consisting of ubiquitin at the N-terminus and ribosomal protein L40 at the C-terminus, and plays a key role in the cell cycle [55]. A previous study reported that this gene is a conserved host factor related to viral replication and pathogenicity of the H5N1 avian influenza virus [56]. In ASFV, the viral ubiquitin-conjugating enzyme (*i.e.* UBCv1, known as I225L) plays a key role in ASFV infection and is reported to be strongly associated with regulating NF-κB signalling [57,58]. Our findings suggest that *UBA52* could be a main host target protein (UBA52) encoded gene by the ASFV protein I225L (UBCv1; a ubiquitin-conjugating enzyme), which potentially modulates many viral mechanisms and cellular functions in the early phase of infection. However, the present findings had a limitation in that the DEGs identified through transcriptome analysis were not validated through laboratory experiments (i.e. ELISA, western blotting). Future studies are needed to verify the expression of major cytokines and key proteins associated with signalling pathways in ASFV infection based on these transcriptomic datasets.

In conclusion, our *in vivo* study highlights the substantial impact of ASFV infection on the gene expression profiles of the host organs from the early stages of infection, to determine the tissue tropism of the virus in pigs. Notably, among other immune-associated genes, *S100A8* was upregulated in multiple organs at an early time-point after viremia (1 and 3 dpi), influencing macrophage activation and the release of inflammatory cytokines. We observed a significant production of critical pro-inflammatory cytokines, including *CXCL2*, *CXCL8*, *CXCL10*, *IL-2*, *IL-4*, *IL-12A*, *IL-33*, *IL-18*, *IL-6*, *IFNG*, *TNFSF13B*, and *TNFSF18*, which play vital roles in antiviral immune responses. KEGG pathway analysis identified the PI3K-Akt signalling pathway in all organs at 1 and 3 dpi, suggesting its potential exploitation by ASFV, to enhance its pathogenesis. In addition, the NF-κB signalling pathway was expressed at 3 d after viremia, which disagreed with previous findings in the *in vitro* experiment with PAMs [13,46]. PPI network analysis revealed that *UBA52* was the top hub gene, suggesting that ubiquitin is an important protein in modulating ASFV mechanisms.

Despite the limitations of this study in verifying the key DEGs and proteins, the overall results demonstrated that the ASFV pathogen enhances its replication and transcription mechanisms to counteract the host immune response, while the host tissues initiate antiviral and immune signalling pathways in response to the viral attack. Although the ASFV inoculation method (intravascular inoculation) in this study did not fully recapitulate natural ASFV infection (oronasal infection), the results serve as a foundational resource for studying ASFV pathogenesis and host immune responses to identify vaccine targets and develop alternative antiviral strategies. Further *in vivo* studies are needed to reflect the natural ASFV infection status via the oronasal route or ingestion of viral infected feed to reveal the transcriptomic responses in each ASFV-infected organ tissue from naturally infected pigs.

## Supplementary Material

Supplemental Material

Supplemental Material

Supplemental Material

Supplemental Material

Supplemental Material

Supplemental Material

Supplemental Material

Supplemental Material

Supplemental Material

Supplemental Material

Supplemental Material

Supplemental Material

Supplemental Material

Supplemental Material

## Data Availability

The transcriptomic datasets generated in this study can be found in the NCBI GEO database, under accession number 230340.
