# Supplementary figures and images for: Transcriptome profiles of organ tissues from pigs experimentally infected with African swine fever virus in early phase of infection
Source: Emerg Microbes Infect. 2024 Jun 7;13(1):2366406. doi: 10.1080/22221751.2024.2366406 (PMC11210422; doi:10.1080/22221751.2024.2366406)

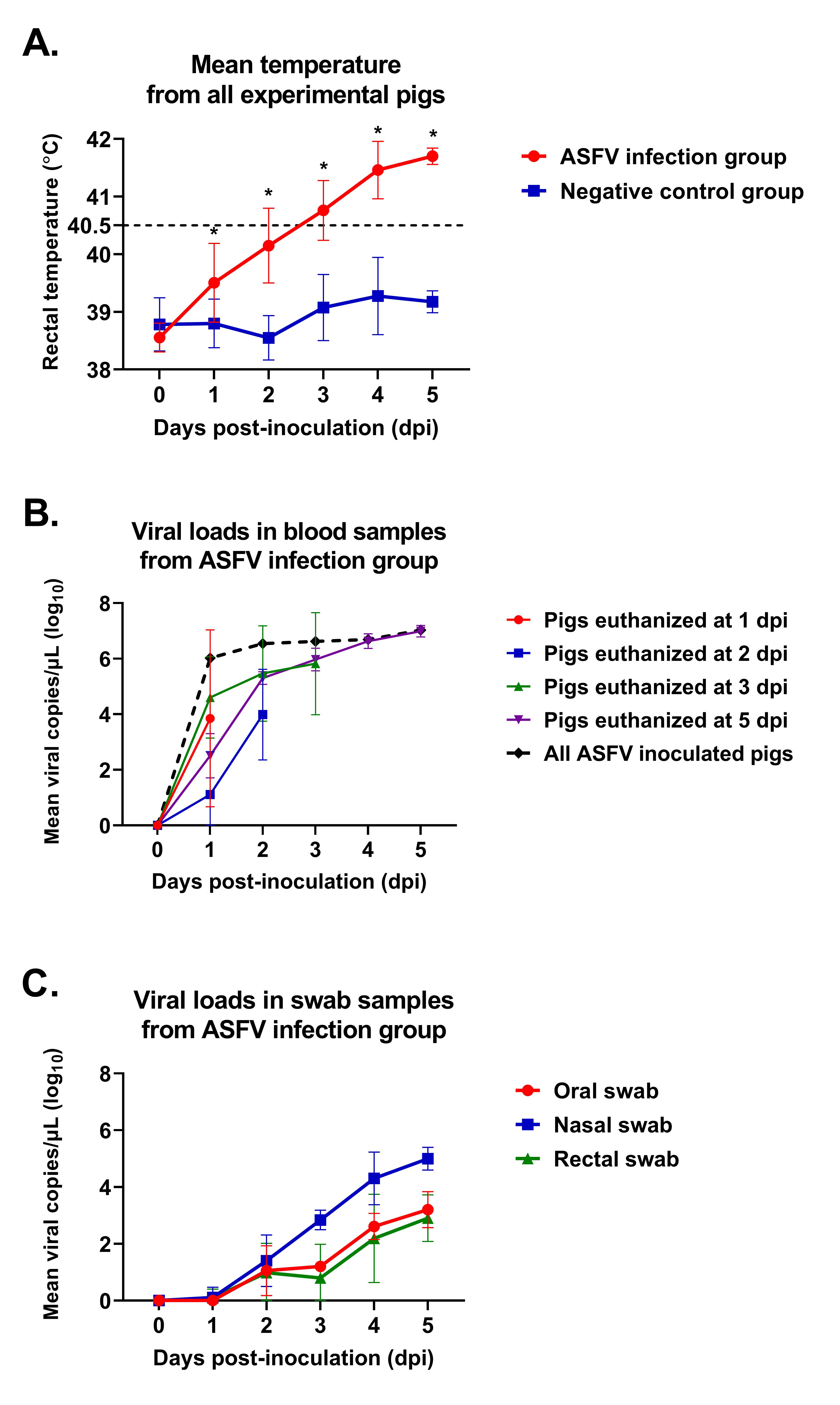

Supplement: Supplemental Material [file TEMI_A_2366406_SM4733.jpg]

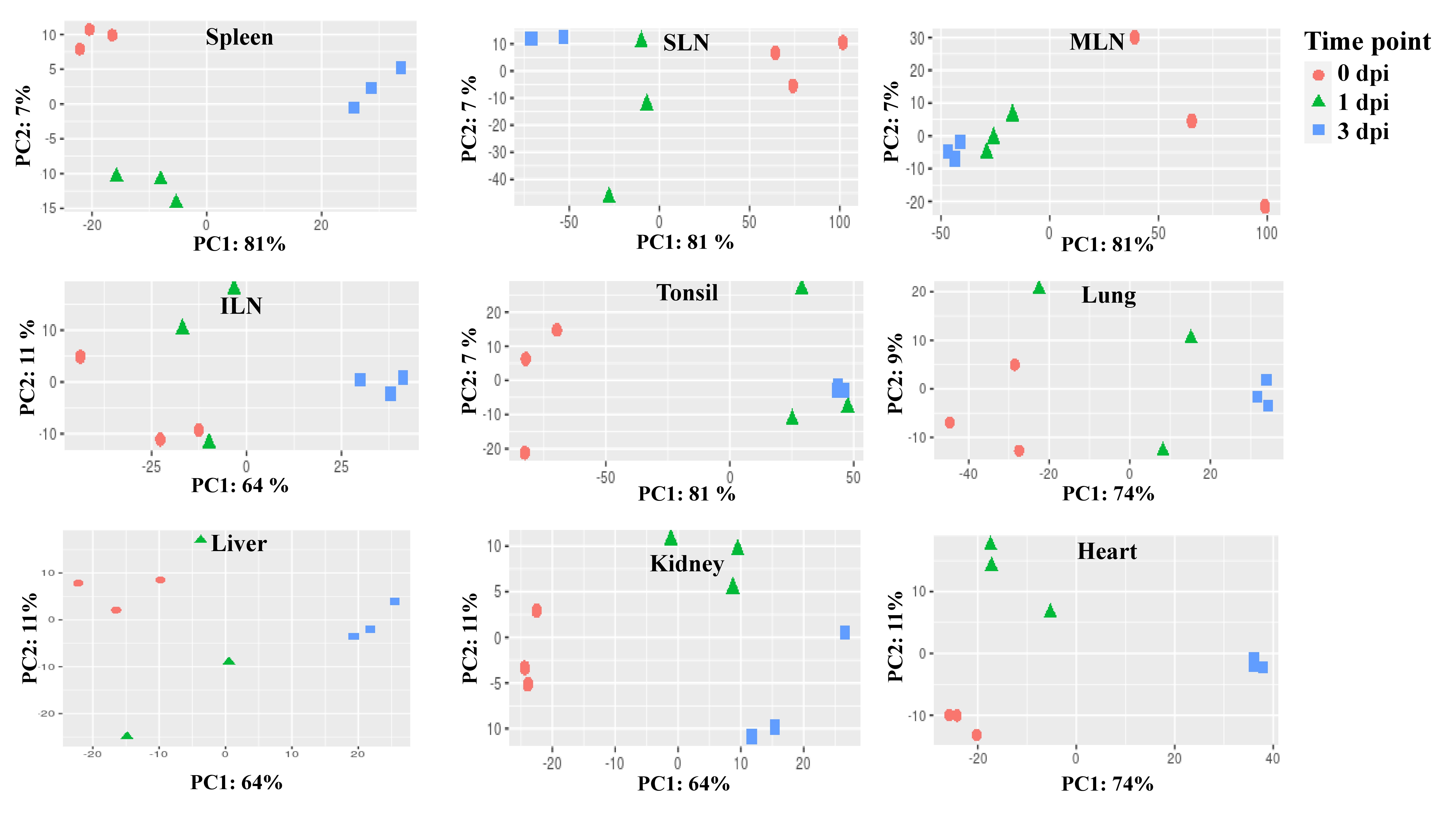

Supplement: Supplemental Material [file TEMI_A_2366406_SM4732.jpg]

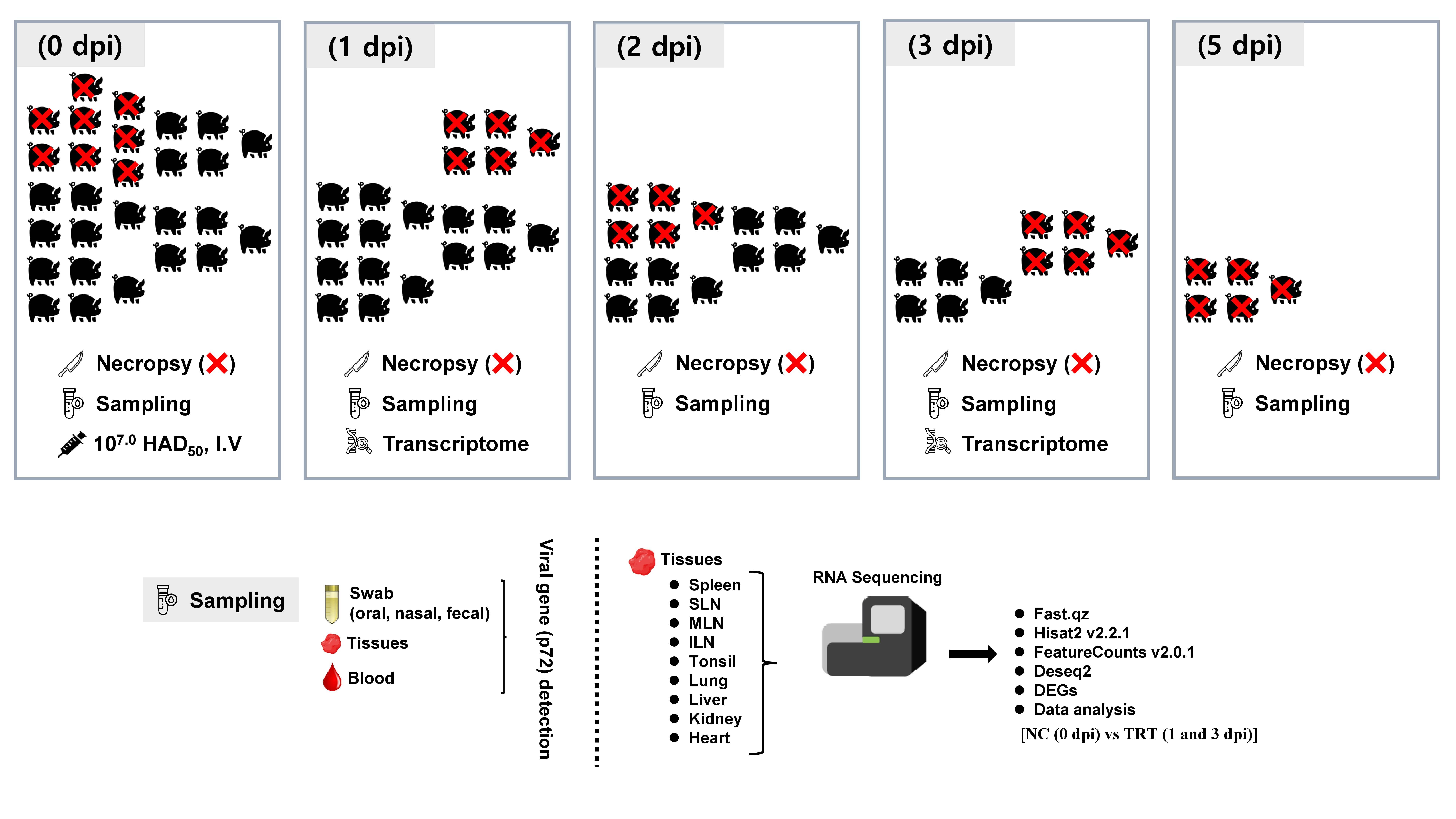

Supplement: Supplemental Material [file TEMI_A_2366406_SM4727.jpg]
