# Supplementary material for: Transcriptome profiles of organ tissues from pigs experimentally infected with African swine fever virus in early phase of infection
Source: Emerg Microbes Infect. 2024 Jun 7;13(1):2366406. doi: 10.1080/22221751.2024.2366406 (PMC11210422; doi:10.1080/22221751.2024.2366406)
Supplement: Supplemental Material [file TEMI_A_2366406_SM4726.docx]

**Supplemental Information for**

Transcriptome profiles of organ tissues from pigs experimentally infected with African swine fever virus in early phase of infection

Sang-Ik Oh ^a,b,*^, Sunirmal Sheet ^a,*^, Vuong Nghia Bui ^c,*^, Duy Tung Dao ^c^, Ngoc Anh Bui ^c^, Tae-Hun Kim ^a,g^, Jihye Cha ^a^, Mi-Rim Park ^a^, Tai-Young Hur ^a^, Young-Hun Jung ^a^, Bumseok Kim ^b^, Hu Suk Lee ^d,e,#^, Ara Cho ^a,#^, Dajeong Lim ^a,f,#^

^a^ National Institute of Animal Science, Rural Development Administration, Wanju 55365, Republic of Korea

^b^ Laboratory of Veterinary Pathology and Biosafety Research Institute, College of Veterinary Medicine, Jeonbuk National University, Iksan 54596, Republic of Korea

^c^ Virology Department, National Institute of Veterinary Research, Hanoi, Vietnam

^d^ International Livestock Research Institute, Hanoi, Vietnam

^e^ College of Veterinary Medicine, Chungnam National University, Daejoen 34134, Republic of Korea

^f^ Department of Animal Resources Science, College of Agriculture and Life Sciences, Chungnam National University, Daejoen 34134, Republic of Korea

^g^ TNT Research. Co., Ltd., R&D center, Sejong-si 30141, Republic of Korea

^*^ S.-I.O, S.S., and V.N.B contributed equally to this work.

^#^ To whom correspondence may be addressed

**E-mail**: hs.lee@cnu.ac.kr, aracho85@korea.kr, or limdj@cnu.ac.kr


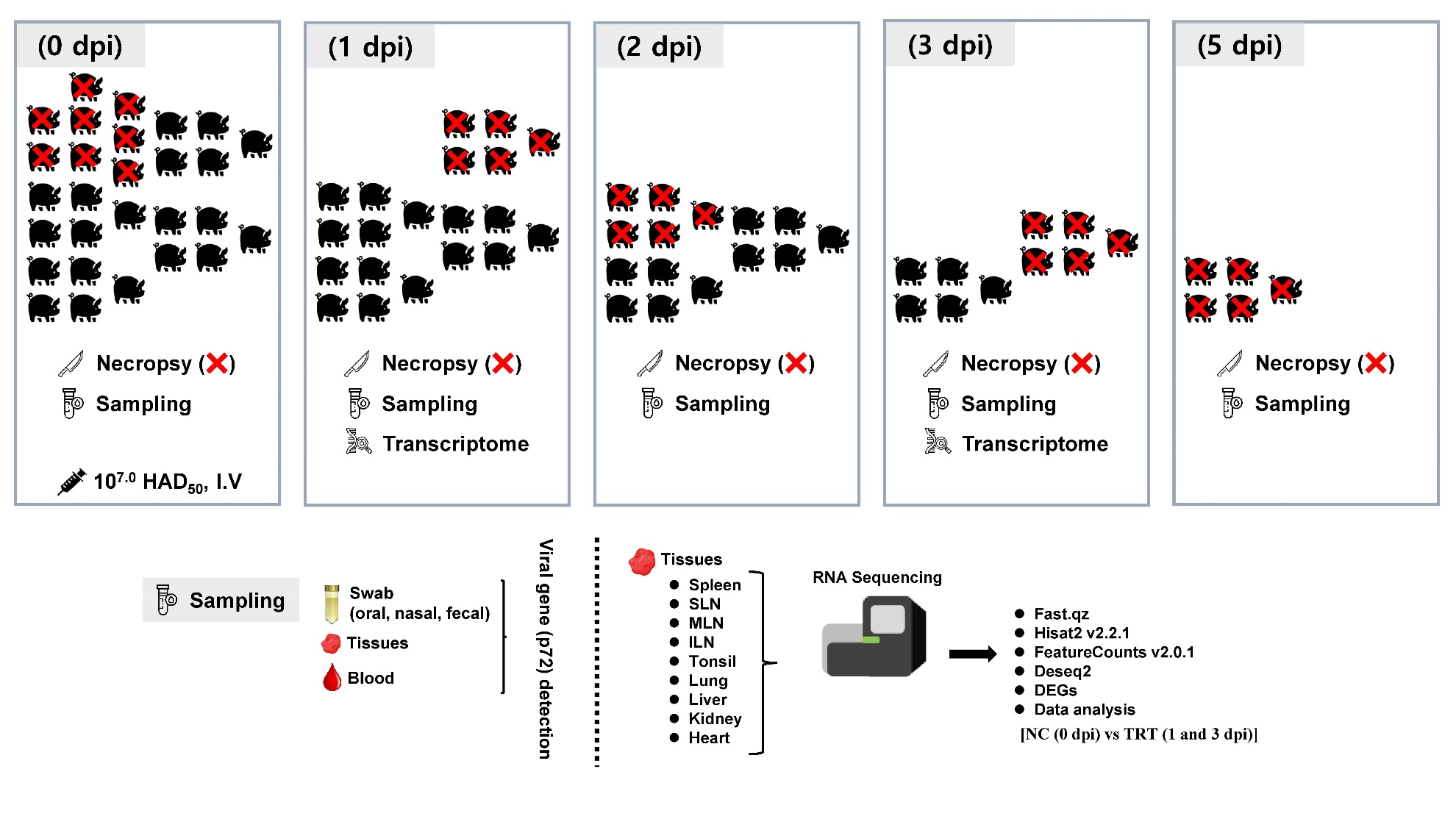


**Fig. S1.** Experimental scheme for the study in the ASFV-infection group (TRT). The tissue samples for the transcriptomic analysis were collected from TRT pigs at 1 and 3 dpi and from NC pigs at 0 dpi.

dpi, days post-inoculation


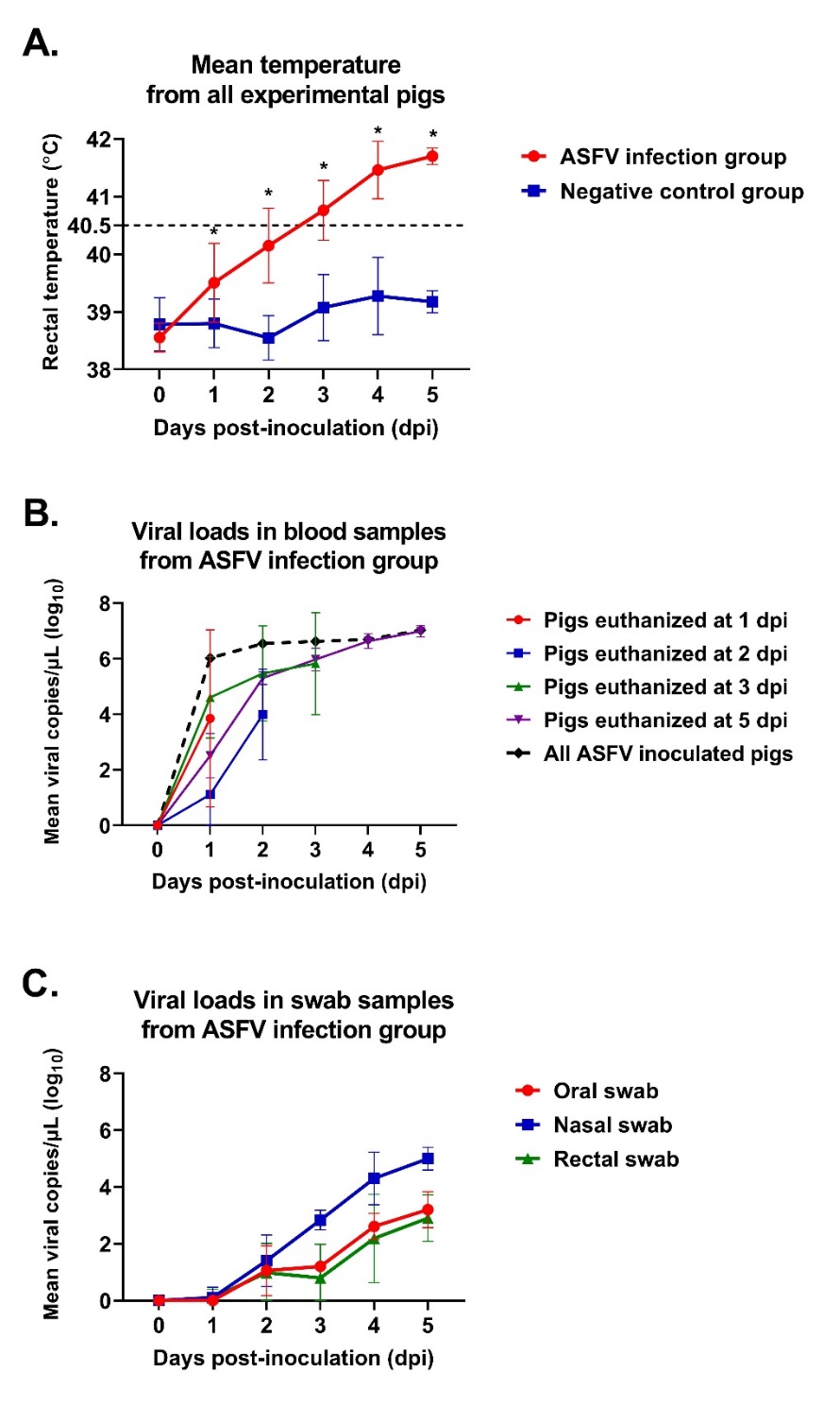


**Fig. S2.** **(A)** Mean rectal temperature in pigs from the ASFV infection (TRT) and negative control groups, according to the period of infection (dpi). ^*^*p*<0.05. Mean number of ASFV viral copies/μL in **(B)** blood samples and **(C)** three swab samples (oral, nasal, and rectal swabs) from the ASFV infection group.

ASFV, African swine fever virus; dpi, days post-infection


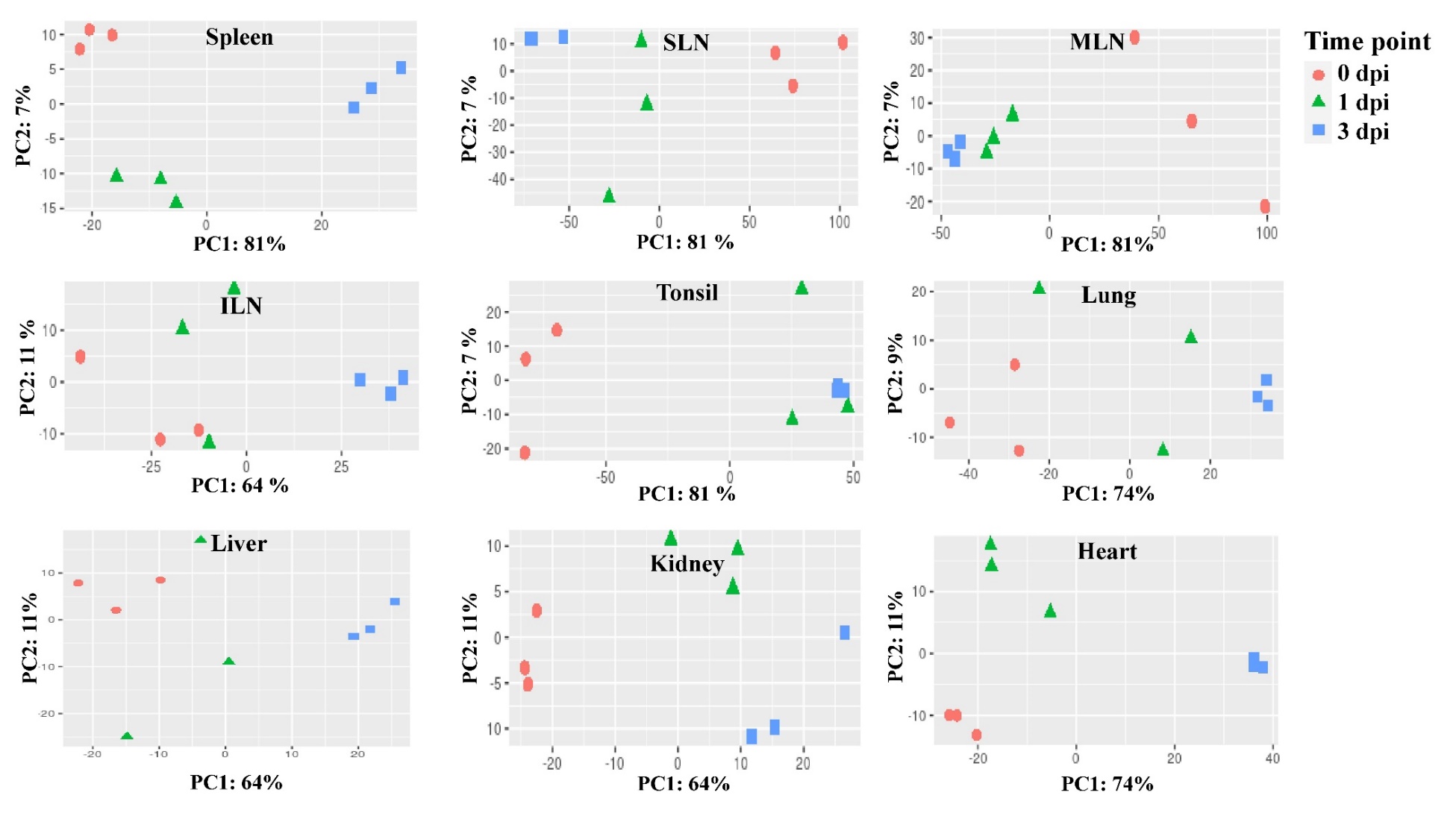


**Fig. S3.** Principal components analysis revealed distinct clustering for each organ tissue and time-point (dpi)

*
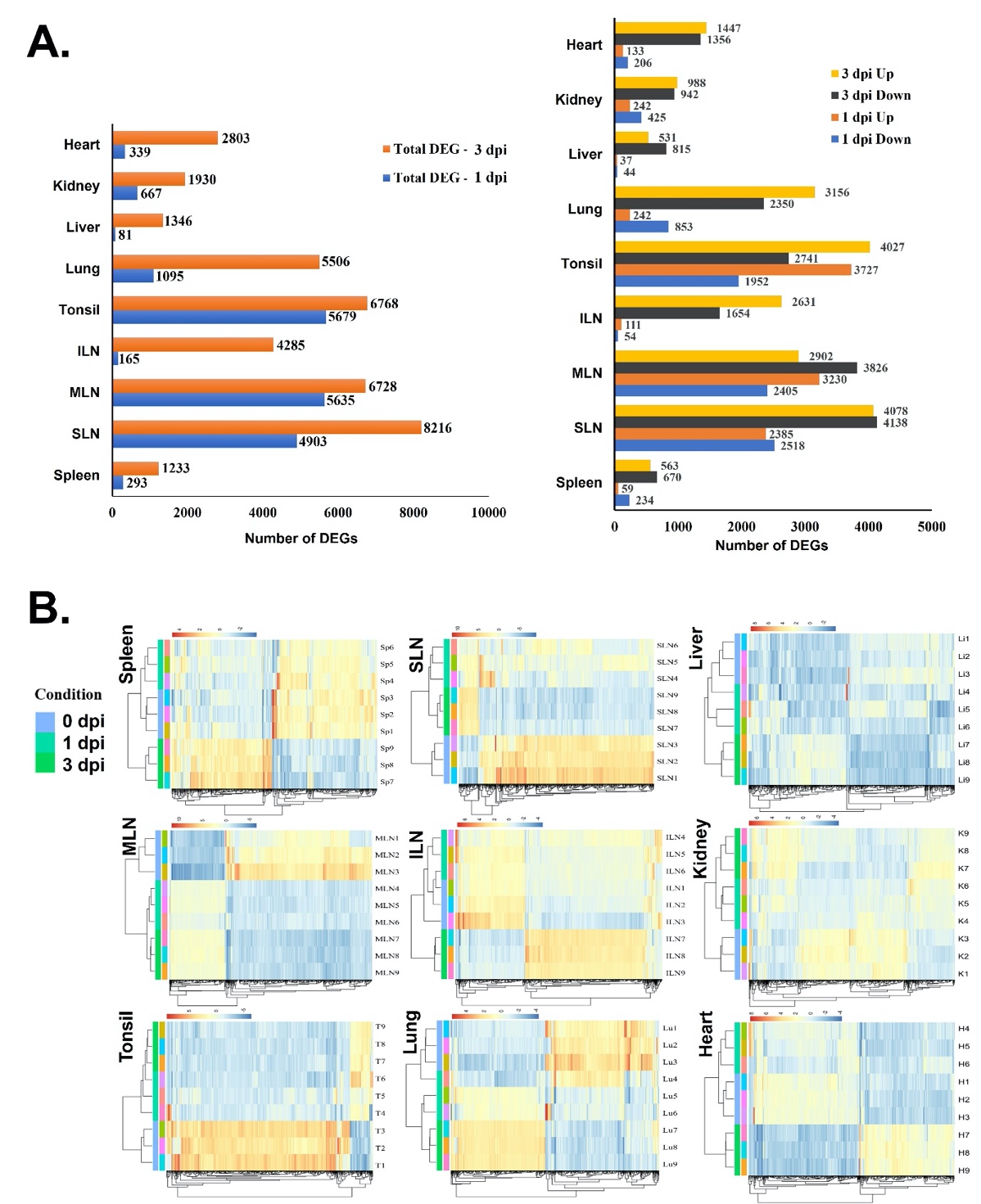
*

**Fig. S4.** DEGs in multiple organ tissues (spleen, SLN, MLN, ILN, tonsils, lungs, liver, kidneys, and heart) from ASFV-infected pigs (TRT group). **(A)** Total number of DEGs in the tissues from TRT pigs, at 1 and 3 dpi, relative to the negative control pigs (p_adj_≤0.05 and log_2_ fold-change≥1.5). **(B)** Hierarchical clustering and heatmap of the Top 500 DEGs in the spleen, SLN, MLN, ILN, tonsil, lung, liver, kidney, and heart tissues from TRT pigs, screened using cut-off criteria of p_adj_≤0.05 and log_2_ fold-change≥1.5.

SLN, submandibular lymph node; MLN, mesenteric lymph node; ILN, inguinal lymph node; DEGs, differentially expressed genes; dpi, days post-inoculation

*
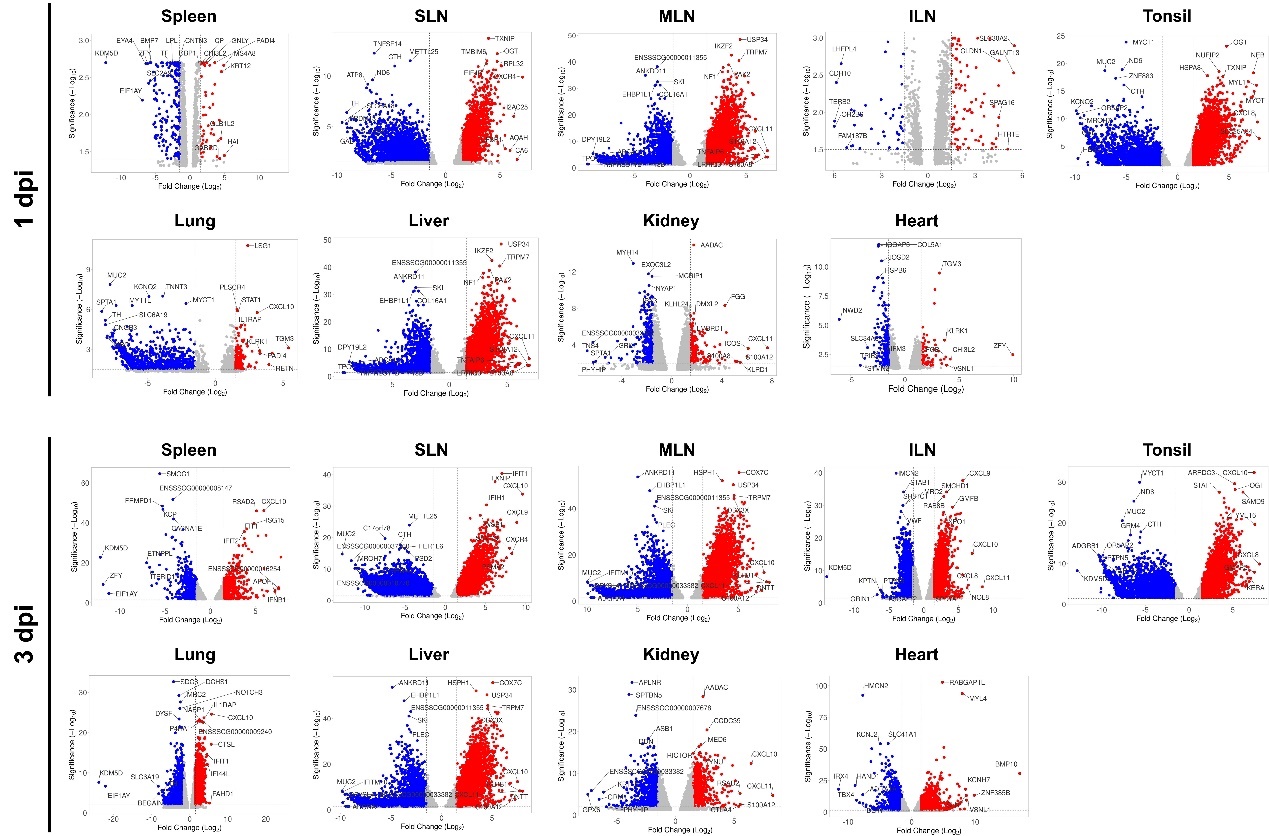
*

**Fig. S5.** Volcano plot visualizing the significantly up- and down-regulated DEGs screened with cut-off criteria of p_adj_≤0.05 and log_2_ fold-change≥1.5 in TRT pigs, at 1 and 3 dpi. The Top 19 DEGs in each tissue, at 1 and 3 dpi, have been shown. The red and blue points indicate up- and down-regulated genes, respectively, while gray points indicate the non-significant genes.

SLN, submandibular lymph node; MLN, mesenteric lymph node; ILN, inguinal lymph node; DEGs, differentially expressed genes; dpi, days post-inoculation


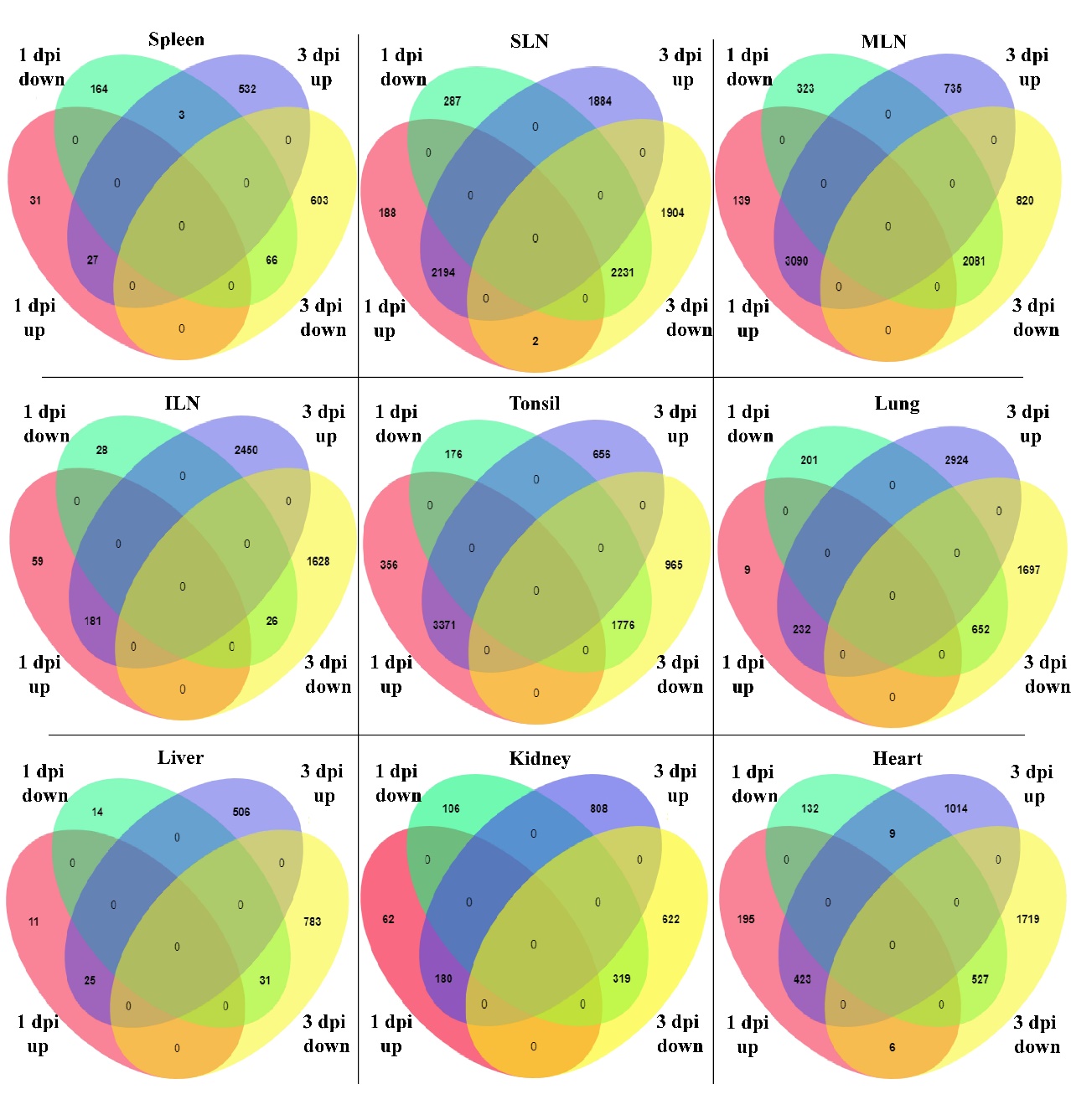


**Fig. S6.** Venn diagrams displaying the overlapped upregulated and downregulated DEGs among the two time-points in individual experimented tissues. SLN - submandibular lymph node; MLN - mesenteric lymph node; ILN - inguinal lymph node.

*
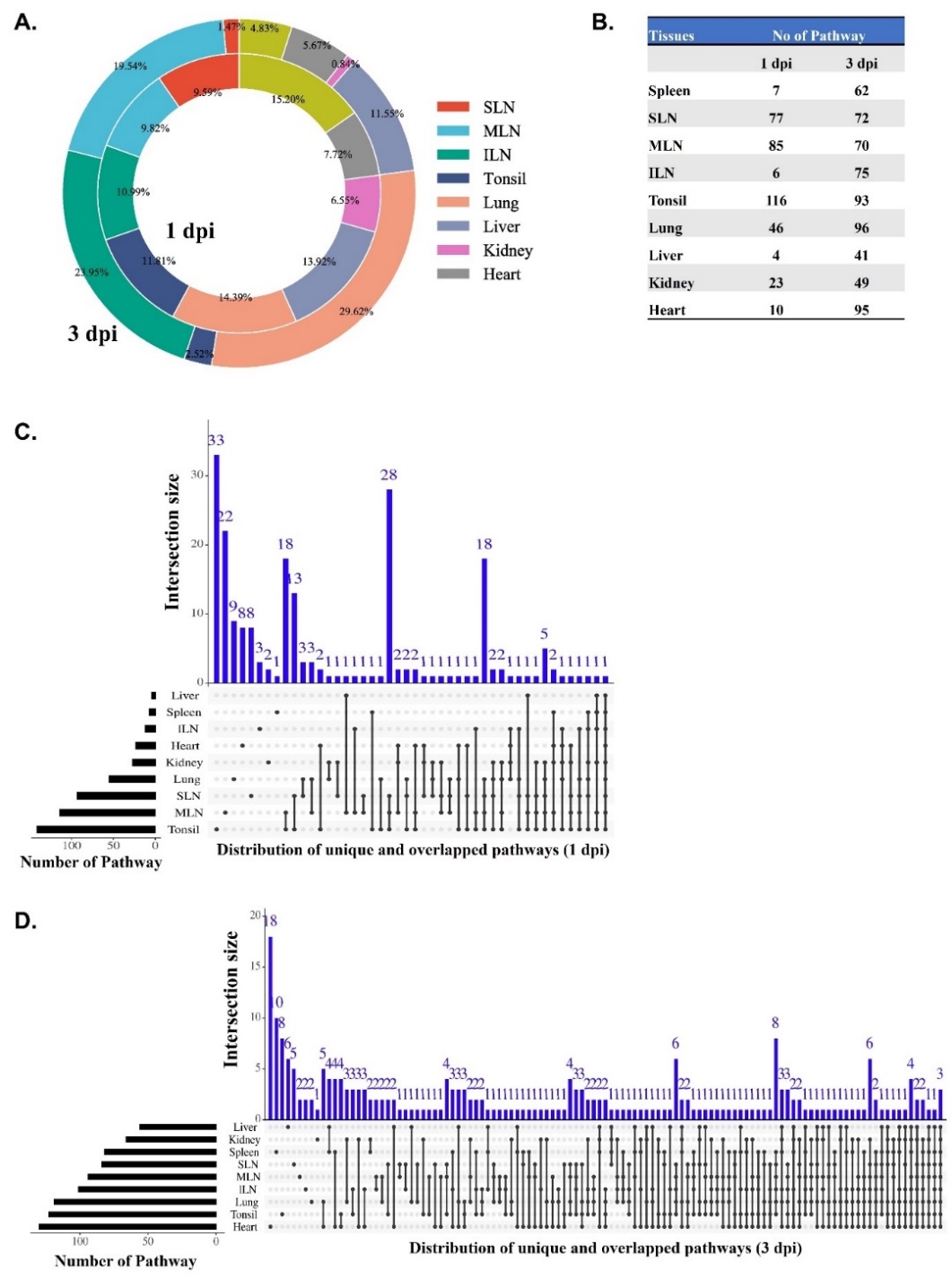
*

**Fig. S7.** Pathway analysis of the genes that displayed expression changes in organ tissues (spleen, SLN, MLN, ILN, tonsils, lungs, liver, kidneys, and heart) obtained from ASFV-inoculated pigs (TRT pigs) euthanized at 1 and 3 dpi. **(A)** The enrichment of the overall pathways has been depicted as percentages for each specific organ. **(B)** The total number of significant pathways (*p*<0.05) enriched in each organ. UpSetR plot providing a visual representation of the unique and overlapping pathways among organ tissues from TRT pigs euthanized at **(C)** 1 and **(D)** 3 dpi. The black arrows indicate the pathways that are shared among all the organ tissues, both at 1 and 3 dpi.

SLN, submandibular lymph node; MLN, mesenteric lymph node; ILN, inguinal lymph node; ASFV, African swine fever virus

**Table S1.** The top 5 up-regulated DEGs identified between ASFV-infected pigs euthanized at 1 and 3 dpi, with the cut-off criteria of log_2_ FC≥1.5 and P_adj_≤0.05

| **Tissue** | **1 dpi** | | |  | **3 dpi** | | |
| --- | --- | --- | --- | --- | --- | --- | --- |
|  | **Gene** | **FC** | **P_adj_** |  | **Gene** | **FC** | **P_adj_** |
| **Spleen** | ***HAL*** | 4.98 | 0.035 |  | ***CXCL11*** | 7.80 | 6.5E-21 |
|  | ***ENSSSCG00000032353*** | 4.89 | 0.044 |  | ***TRIM69*** | 7.56 | 3.5E-06 |
|  | ***MS4A8*** | 4.87 | 0.002 |  | ***ENSSSCG00000016254*** | 7.54 | 8.3E-07 |
|  | ***KRT12*** | 4.55 | 0.002 |  | ***APOH*** | 7.15 | 1.0E-05 |
|  | ***GLB1L2*** | 4.46 | 0.020 |  | ***IFNB1*** | 7.14 | 5.2E-05 |
| **SLN** | ***CXCR4*** | 7.09 | 1.3E-10 |  | ***CXCL10*** | 9.62 | 1.5E-34 |
|  | ***CA6*** | 6.58 | 0.017 |  | ***CXCL9*** | 8.88 | 1.4E-25 |
|  | ***H2AC25*** | 6.27 | 9.5E-07 |  | ***CXCR4*** | 8.27 | 1.1E-15 |
|  | ***AOAH*** | 5.92 | 0.002 |  | ***SAMD9*** | 8.00 | 2.4E-17 |
|  | ***UFSP1*** | 5.49 | 0.001 |  | ***PSMA2*** | 7.61 | 2.5E-14 |
| **MLN** | ***S100A12*** | 6.90 | 2.0E-05 |  | ***DNTT*** | 8.04 | 6.4E-09 |
|  | ***CXCL11*** | 6.88 | 4.2E-08 |  | ***KLRB1*** | 7.80 | 5.0E-09 |
|  | ***S100A8*** | 6.75 | 1.8E-05 |  | ***CXCL11*** | 7.74 | 4.9E-09 |
|  | ***TNFAIP6*** | 6.10 | 2.5E-06 |  | ***CXCL10*** | 7.53 | 3.9E-13 |
|  | ***LRRIQ3*** | 5.95 | 1.1E-05 |  | ***S100A12*** | 7.29 | 2.5E-05 |
| **ILN** | ***SLC30A2*** | 5.49 | 0.001 |  | ***CXCL11*** | 8.42 | 1.0E-05 |
|  | ***GALNT13*** | 5.46 | 0.003 |  | ***CXCL10*** | 6.98 | 5.6E-16 |
|  | ***HTR1E*** | 5.08 | 0.031 |  | ***CXCL8*** | 6.40 | 2.9E-06 |
|  | ***CLDN1*** | 4.52 | 0.002 |  | ***NOL8*** | 6.23 | 2.1E-05 |
|  | ***SPAG16*** | 4.51 | 0.012 |  | ***SPDYA*** | 5.68 | 6.6E-04 |
| **Tonsils** | ***SLC25A34*** | 7.95 | 4.0E-06 |  | ***CXCL8*** | 8.03 | 3.0E-09 |
|  | ***CXCL8*** | 7.80 | 1.5E-08 |  | ***ZFYVE16*** | 7.52 | 1.2E-17 |
|  | ***MYL11*** | 7.68 | 5.2E-14 |  | ***GMPR2*** | 7.48 | 1.5E-07 |
|  | ***MYOT*** | 7.45 | 4.6E-13 |  | ***CXCL10*** | 7.46 | 1.9E-29 |
|  | ***NEB*** | 7.37 | 4.9E-16 |  | ***KERA*** | 7.03 | 3.0E-06 |
| **Lungs** | ***TGM3*** | 5.39 | 0.001 |  | ***CXCL10*** | 5.77 | 2.6E-25 |
|  | ***RETN*** | 3.94 | 0.002 |  | ***CTSL*** | 5.64 | 7.6E-18 |
|  | ***PADI4*** | 3.29 | 0.014 |  | ***FAHD1*** | 5.20 | 3.4E-03 |
|  | ***KLRK1*** | 3.27 | 0.002 |  | ***ENSSSCG00000004180*** | 4.68 | 8.9E-13 |
|  | ***CXCL10*** | 3.10 | 0.001 |  | ***IFIT1*** | 4.51 | 2.9E-15 |
| **Liver** | ***MMP8*** | 5.38 | 7.4E-05 |  | ***ENSSSCG00000033183*** | 7.12 | 2.6E-07 |
|  | ***TGM3*** | 4.99 | 1.4E-11 |  | ***CHI3L2*** | 7.08 | 2.5E-07 |
|  | ***CHI3L2*** | 4.86 | 1.1E-02 |  | ***CCDC33*** | 7.02 | 3.3E-04 |
|  | ***LCN2*** | 4.58 | 5.6E-04 |  | ***ENSSSCG00000016254*** | 6.99 | 6.3E-05 |
|  | ***SH2D4B*** | 3.70 | 3.3E-08 |  | ***NYAP2*** | 5.74 | 4.3E-06 |
| **Kidneys** | ***CXCL11*** | 7.66 | 0.001 |  | ***CXCL11*** | 8.29 | 2E-05 |
|  | ***ICOS*** | 6.12 | 0.001 |  | ***CXCL10*** | 6.43 | 4E-13 |
|  | ***S100A12*** | 6.01 | 0.002 |  | ***S100A12*** | 5.52 | 3E-03 |
|  | ***KLRD1*** | 5.48 | 0.008 |  | ***RSAD2*** | 5.50 | 5E-06 |
|  | ***CXCL11*** | 5.24 | 0.007 |  | ***CTLA4*** | 5.37 | 8E-03 |
| **Heart** | ***ZFY*** | 9.95 | 0.003 |  | ***BMP10*** | 9.97 | 2.6E-31 |
|  | ***EIF1AY*** | 9.59 | 0.061 |  | ***ZNF385B*** | 9.89 | 4.8E-14 |
|  | ***KDM5D*** | 9.19 | 0.073 |  | ***VSNL1*** | 8.69 | 3.2E-10 |
|  | ***ENSSSCG00000008998*** | 4.79 | 0.047 |  | ***SLN*** | 8.63 | 6.9E-14 |
|  | ***CHI3L2*** | 4.17 | 0.008 |  | ***KCNH7*** | 8.49 | 7.6E-19 |

FC, fold-change; SLN, submandibular lymph node; MLN, mesenteric lymph node; ILN, inguinal lymph node; dpi, days post-inoculation; ASFV, African swine fever virus

**Table S2.** Gene Ontology (BP) and KEGG pathways significantly (*p* < 0.05) enriched by DEGs of ASFV infected tissues at 1 dpi

|  | **Heart** | | **ILN** | **Kidney** | | **Liver** | **Lung** | |
| --- | --- | --- | --- | --- | --- | --- | --- | --- |
| GO - BP | negative regulation of T cell activation  astrocyte development  mitotic spindle assembly  positive regulation of natural killer cell mediated cytotoxicity  defense response to Gram-positive bacterium  response to bacterium  negative regulation of fat cell differentiation  negative regulation of interleukin-8 production  antimicrobial humoral immune response mediated by antimicrobial peptide  gamma-delta T cell differentiation and more (13) | | positive regulation of interleukin-8 production  regulation of behavior  establishment of localization in cell  potassium ion transmembrane transport  chloride transmembrane transport  Fanconi anemia pathway  Cytokine-cytokine receptor interaction  Neuroactive ligand-receptor interaction  Axon guidance  Complement and coagulation cascades and more (7) | immune response  defense response to virus  odontogenesis of dentin-containing tooth  regulation of cell adhesion  negative regulation of myoblast differentiation  negative regulation of bone mineralization  endodermal cell differentiation  response to cold  positive regulation of interferon-gamma production  response to dietary excess and more (41) | | leukocyte migration involved in inflammatory response  negative regulation of endopeptidase activity  astrocyte development  peptidyl-cysteine S-nitrosylation  peptide cross-linking  ventricular system development  collagen catabolic process  response to lipopolysaccharide  negative regulation of interleukin-10 production  cellular response to insulin stimulus and more (13) | cellular response to light stimulus  hormone metabolic process  regulation of cardiac muscle contraction  male gonad development  spermatogenesis  antimicrobial humoral immune response mediated by antimicrobial peptide  synapse organization  long-term synaptic potentiation  neuromuscular synaptic transmission  regulation of long-term neuronal synaptic plasticity and more (76) | |
| KEGG Pathway | ECM-receptor interaction  Protein digestion and absorption  Focal adhesion  Human papillomavirus infection  Th1 and Th2 cell differentiation  PI3K-Akt signaling pathway  Complement and coagulation cascades  Primary immunodeficiency  Th17 cell differentiation  Hematopoietic cell lineage | | Fanconi anemia pathway  Cytokine-cytokine receptor interaction  Neuroactive ligand-receptor interaction  Axon guidance  Complement and coagulation cascades  IL-17 signaling pathway  Synaptic vesicle cycle  Nicotine addiction  Rheumatoid arthritis  PI3K-Akt signaling pathway | Cardiac muscle contraction  Calcium signaling pathway  GABAergic synapse  Neuroactive ligand-receptor interaction  Cocaine addiction  Primary immunodeficiency  Coronavirus disease - COVID-19  PI3K-Akt signaling pathway  IL-17 signaling pathway  Oxytocin signaling pathway and more (12) | | Glycerophospholipid metabolism  Metabolic pathways  PI3K-Akt signaling pathway  IL-17 signaling pathway | Complement and coagulation cascades, Cytokine-cytokine receptor interaction, Chemical carcinogenesis - receptor activation, Other types of O-glycan biosynthesis, Adrenergic signaling in cardiomyocytes, Estrogen signaling pathway, ABC transporters, Aldosterone synthesis and secretion, Viral protein interaction with cytokine and cytokine receptor, PI3K-Akt signaling pathway and more (37) | |
|  | **MLN** | **Muscle** | | **SLN** | **Spleen** | | | **Tonsil** |
| GO - BP | positive regulation of B cell apoptotic process  negative regulation of hydrogen peroxide-induced neuron death  negative regulation of myeloid dendritic cell activation  regulation of response to wounding  negative regulation of chronic inflammatory response to antigenic stimulus  negative regulation of MHC class II biosynthetic process  negative regulation of cytokine activity  cellular response to hepatocyte growth factor stimulus  positive regulation of plasma cell differentiation  negative regulation of heterotypic cell-cell adhesion and more (102) | viral RNA genome packaging  positive regulation by host of viral process  viral release from host cell  pyruvate metabolic process  gluconeogenesis  regulation of centrosome duplication  cell differentiation  negative regulation of transcription from RNA polymerase II promoter  zinc II ion transport  negative regulation of gene expression and more (42) | | negative regulation of neuron apoptotic process  positive regulation by host of viral transcription  motile cilium assembly  positive regulation by host of viral genome replication  negative regulation of neuron differentiation  positive regulation of cytolysis  pore complex assembly  positive regulation of mRNA splicing, via spliceosome  rhythmic process  smooth muscle tissue development and more (124) | positive regulation of cell proliferation  response to glucose  iron ion transport  heterophilic cell-cell adhesion via plasma membrane cell adhesion molecules  positive regulation of interleukin-12 production  negative regulation of canonical Wnt signaling pathway  inflammatory response  positive regulation of protein localization to plasma membrane  chemokine-mediated signaling pathway  bicellular tight junction assembly and more (22) | | | cell morphogenesis  skeletal system development  behavioral fear response  neuron migration  negative regulation of cytokine production  blastocyst formation  regulation of protein phosphorylation  morphogenesis of an epithelium  toll-like receptor signaling pathway  tricarboxylic acid cycle and more (117) |
| KEGG Pathway | Metabolic pathways  Hedgehog signaling pathway  Protein digestion and absorption  Pathways in cancer  IL-17 signaling pathway  Thermogenesis  Oxidative phosphorylation,  PI3K-Akt signaling pathway, TGF-beta signaling pathway, Pathways of neurodegeneration - multiple diseases and more (75) | Human papillomavirus infection  Thermogenesis  Basal transcription factors  p53 signaling pathway  Gastric acid secretion  PI3K-Akt signaling pathway  Autophagy - animal  Acute myeloid leukemia  Insulin resistance  Non-alcoholic fatty liver disease and more (30) | | PI3K-Akt signaling pathway  Ferroptosis  Cardiac muscle contraction  Adrenergic signaling in cardiomyocytes  Cell adhesion molecules  Circadian entrainment  Thermogenesis  Long-term potentiation  Synaptic vesicle cycle  Retrograde endocannabinoid signaling and more (77) | PPAR signaling pathway  Histidine metabolism  ECM-receptor interaction  IL-17 signaling pathway  PI3K-Akt signaling pathway  Focal adhesion  Neuroactive ligand-receptor interaction  Cytokine-cytokine receptor interaction  AMPK signaling pathway  Nicotine addiction | | | TGF-beta signaling pathway  Morphine addiction  Complement and coagulation cascades  Pancreatic cancer  Mitophagy - animal  Type II diabetes mellitus  Renal cell carcinoma  Amino sugar and nucleotide sugar metabolism  Choline metabolism in cancer  PI3K-Akt signaling pathway and more (106) |

SLN - submandibular lymph node; MLN - mesenteric lymph node; ILN - inguinal lymph node

**Table S3.** Gene Ontology (BP) and KEGG pathways significantly (*p* < 0.05) enriched by DEGs of ASFV infected tissues at 3 dpi

|  | **Heart** | **ILN** | **Kidney** | **Liver** | **Lung** |
| --- | --- | --- | --- | --- | --- |
| GO - BP | Positive regulation of transcription from RNA polymerase II promoter, immune response, intracellular signal transduction, cell differentiation, cell adhesion, positive regulation of gene expression, innate immune response, inflammatory response, cellular response to lipopolysaccharide, defense response to bacterium and more (144) | Positive regulation of transcription from RNA polymerase II promoter, protein transport, ubiquitin-dependent protein catabolic process, negative regulation of gene expression, positive regulation of apoptotic process, in utero embryonic development, DNA repair  extracellular matrix organization, regulation of cell cycle, cell division and more (90) | detection of molecule of bacterial origin, positive regulation of cytolysis, positive regulation of respiratory burst involved in inflammatory response, positive regulation of chemokine production, lipopolysaccharide transport  negative regulation of viral genome replication, interleukin-27-mediated signaling pathway, neural crest cell development, positive regulation of tumor necrosis factor production, macrophage activation involved in immune response and more (75) | antimicrobial humoral immune response mediated by antimicrobial peptide, positive regulation of GTPase activity, positive regulation of NIK/NF-kappaB signaling, positive regulation of interferon-beta production, negative chemotaxis, negative regulation of cellular component movement, negative regulation of cell adhesion molecule production, inflammatory response, outflow tract morphogenesis, aortic valve morphogenesis and more (82) | positive regulation of interferon-alpha production  regulation of ion transmembrane transport  positive regulation of transcription from RNA polymerase II promoter  pituitary gland development  peptidyl-serine phosphorylation  calcium ion import  cellular response to calcium ion  fibroblast growth factor receptor signaling pathway  neuron migration  cardiac conduction and more (98) |
| KEGG Pathway | Pathways in cancer, Pathways of neurodegeneration-multiple diseases, PI3K-Akt signaling pathway, MAPK signaling pathway, Human papillomavirus infection, Cytokine-cytokine receptor interaction, Calcium signaling pathway, cAMP signaling pathway, Chemokine signaling pathway, Focal adhesion and more (85) | Pathways of neurodegeneration - multiple diseases, Pathways in cancer, Alzheimer disease, Amyotrophic lateral sclerosis, Huntington disease, Human papillomavirus infection, Prion disease, Parkinson disease, MAPK signaling pathway, Endocytosis and more (64) | PI3K-Akt signaling pathway, NF-kappa B signaling pathway, IL-17 signaling pathway, Toll-like receptor signaling pathway, Alcoholism, C-type lectin receptor signaling pathway, Cytokine-cytokine receptor interaction, Hedgehog signaling pathway, Complement and coagulation cascades, Chemokine signaling pathway and more (57) | ECM-receptor interaction  Human papillomavirus infection, PI3K-Akt signaling pathway, Pathways in cancer, Focal adhesion, Cytokine-cytokine receptor interaction, Fluid shear stress and atherosclerosis, Axon guidance, Protein digestion and absorption, Insulin signaling pathway, and more (46) | Spinocerebellar ataxia  MAPK signaling pathway  Pathways in cancer  Human papillomavirus infection  Prion disease  Focal adhesion  Dopaminergic synapse  Calcium signaling pathway  Alzheimer disease  Pathways of neurodegeneration - multiple diseases and more (86) |
|  | **MLN** | **Muscle** | **SLN** | **Spleen** | **Tonsil** |
| GO - BP | negative regulation of cytokine production involved in immune response, positive regulation of B cell apoptotic process, negative regulation of cytokine activity, cellular response to hepatocyte growth factor stimulus, positive regulation of MHC class II biosynthetic process  positive regulation of receptor activity  cellular response to lipopolysaccharide  branching involved in labyrinthine layer morphogenesis  positive regulation of cytokine production  positive regulation of immunoglobulin production and more (136) | positive regulation by host of viral process  viral RNA genome packaging  viral release from host cell  pyruvate metabolic process  gluconeogenesis  transforming growth factor beta receptor signaling pathway  cardiac conduction  negative regulation of gene expression  Rho protein signal transduction  zinc II ion transport and more (66) | embryonic cranial skeleton morphogenesis  proximal/distal pattern formation  gamma-aminobutyric acid signaling pathway  positive regulation by host of viral genome replication  regulation of dopamine secretion  negative regulation of viral genome replication  intermediate filament organization  positive regulation of monocyte chemotaxis  positive regulation of macrophage activation  positive regulation of toll-like receptor 4 signaling pathway and more (138) | extracellular matrix organization  negative regulation of viral genome replication  defense response to virus  neutrophil chemotaxis  proteolysis involved in cellular protein catabolic process  collagen catabolic process  protein folding  cellular response to virus  immune response  positive regulation of cell migration and more (91) | positive regulation of MHC class II biosynthetic process  positive regulation of receptor activity  negative regulation of vascular smooth muscle cell proliferation  cellular response to hepatocyte growth factor stimulus  negative regulation of endothelial cell apoptotic process  negative regulation of B cell proliferation  response to glucocorticoid  negative regulation of membrane protein ectodomain proteolysis  positive regulation of cell cycle  negative regulation of interleukin-6 production and more (139) |
| KEGG Pathway | mRNA surveillance pathway  Vasopressin-regulated water reabsorption  Nucleotide metabolism  Citrate cycle (TCA cycle)  Neurotrophin signaling pathway  IL-17 signaling pathway  Circadian rhythm  Gastric acid secretion  NF-kappa B signaling pathway  Chemokine signaling pathway and more (64) | Basal cell carcinoma  Gastric cancer  Phospholipase D signaling pathway  AMPK signaling pathway  Choline metabolism in cancer  Cushing syndrome  Bacterial invasion of epithelial cells  NF-kappa B signaling pathway  Chemokine signaling pathway  T cell receptor signaling pathway and more (93) | Protein digestion and absorption  Estrogen signaling pathway  HIF-1 signaling pathway  NF-kappa B signaling pathway  Apelin signaling pathway  Aldosterone synthesis and secretion  Chemokine signaling pathway Biosynthesis of amino acids  Serotonergic synapse  PI3K-Akt signaling pathway and more (62) | Cytokine-cytokine receptor interaction, PI3K-Akt signaling pathway, Viral protein interaction with cytokine and cytokine receptor, IL-17 signaling pathway, Chemokine signaling pathway, Toll-like receptor signaling pathway, NF-kappa B signaling pathway, T cell receptor signaling pathway, Complement and coagulation cascades, ECM-receptor interaction, and more (53) | TGF-beta signaling pathway, IL-17 signaling pathway, Chemokine signaling pathway, NF-kappa B signaling pathway, Glutamatergic synapse, Mineral absorption, Chemical carcinogenesis - reactive oxygen species, Retrograde endocannabinoid signaling, Nucleocytoplasmic transport, Oocyte meiosis and more (84) |

SLN - submandibular lymph node; MLN - mesenteric lymph node; ILN - inguinal lymph node

**Table S4.** Top 10 hub genes identified from the protein-protein interaction network

| **Hub genes** | **Closeness centrality** | **Degree** | **Neighborhood connectivity** | **Undirected edges (*n*)** |
| --- | --- | --- | --- | --- |
| *UBA52* | 0.474289171 | 58 | 35.13793103 | 58 |
| *RPS3* | 0.50524109 | 54 | 38.7962963 | 54 |
| *PSMA4* | 0.538784067 | 54 | 38.87037037 | 54 |
| *PSMA3* | 0.598793363 | 52 | 39.73076923 | 52 |
| *BBS10* | 0.512820513 | 52 | 37.15384615 | 52 |
| *UBD* | 0.523921569 | 51 | 36.90196078 | 51 |
| *PSMA7* | 0.676862745 | 51 | 41.84313725 | 51 |
| *PSMA8* | 0.676862745 | 51 | 41.84313725 | 51 |
| *NDUFA1* | 0.768163265 | 50 | 42.22 | 50 |
| *CCT5* | 0.571428571 | 49 | 38.81632653 | 49 |

Dataset S1 (separate file). Total differential expression genes (DEGs) at cut-off of q < 0.05 and log2 fold change ≥ 1.5 at 1 dpi time-point.

Dataset S2 (separate file). Total differential expression genes (DEGs) at cut-off of q < 0.05 and log2 fold change ≥ 1.5 at 3 dpi time-point.

Dataset S3 (separate file). Overlapping DEGs between nine tissues at 1 dpi and 3 dpi time-points.

Dataset S4 (separate file). Expression profile of immune related genes, cytokines and their receptors in ASFV-infected host tissues at 1 dpi and 3 dpi

Dataset S5 (separate file). GO and KEGG pathway enrichment analyses of DEGs in nine tissues infected with ASFV at 1 and 3 dpi.

Dataset S6 (separate file). Biological pathway enrichment analysis of genes from the three modules of the PPI network.
